# Recovery of Fatty Acids from Mineralogic Mars Analogs by TMAH Thermochemolysis for the Sample Analysis at Mars Wet Chemistry Experiment on the Curiosity Rover

**DOI:** 10.1089/ast.2018.1819

**Published:** 2019-03-27

**Authors:** Amy J. Williams, Jennifer Eigenbrode, Melissa Floyd, Mary Beth Wilhelm, Shane O'Reilly, Sarah Stewart Johnson, Kathleen L. Craft, Christine A. Knudson, Slavka Andrejkovičová, James M.T. Lewis, Arnaud Buch, Daniel P. Glavin, Caroline Freissinet, Ross H. Williams, Cyril Szopa, Maëva Millan, Roger E. Summons, Amy McAdam, Kathleen Benison, Rafael Navarro-González, Charles Malespin, Paul R. Mahaffy

**Affiliations:** ^1^ Department of Physics, Astronomy, and Geosciences, Towson University, Towson, Maryland, USA.; ^2^ Center for Research and Exploration in Space Sciences and Technology/University of Maryland Baltimore County, Baltimore, Maryland, USA.; ^3^ Space Science Exploration Division (Code 690), NASA Goddard Space Flight Center, Greenbelt, Maryland, USA.; ^4^ NASA Ames Research Center, Mountain View, California, USA.; ^5^ Department of Earth, Atmospheric and Planetary Sciences, Massachusetts Institute of Technology, Cambridge, Massachusetts, USA.; ^6^ School of Earth Sciences, University College Dublin, Dublin, Ireland.; ^7^ Department of Biology, Georgetown University, Washington, DC, USA.; ^8^ Johns Hopkins University Applied Physics Laboratory, Laurel, Maryland, USA.; ^9^ Center for Research and Exploration in Space Sciences and Technology/University of Maryland College Park, College Park, Maryland, USA.; ^10^ Universities Space Research Association, Columbia, Maryland, USA.; ^11^ Laboratoire de Génie des Procédés et Matériaux, CentraleSupelec, Gif sur Yvette, France.; ^12^ CNRS–UVSQ Laboratoire Atmosphères Milieux Observations Spatiales LATMOS, Guyancourt, France.; ^13^ Department of Geology and Geography, West Virginia University, Morgantown, West Virginia, USA.; ^14^ Instituto de Ciencias Nucleares, Universidad Nacional Autonoma de Mexico, Circuito Exterior, Ciudad Universitaria, Ciudad de Mexico, Mexico.

**Keywords:** Mars, Sample Analysis at Mars (SAM) instrument, Molecular biosignatures, TMAH, MSL, FAME

## Abstract

The Mars Curiosity rover carries a diverse instrument payload to characterize habitable environments in the sedimentary layers of Aeolis Mons. One of these instruments is Sample Analysis at Mars (SAM), which contains a mass spectrometer that is capable of detecting organic compounds via pyrolysis gas chromatography mass spectrometry (py-GC-MS). To identify polar organic molecules, the SAM instrument carries the thermochemolysis reagent tetramethylammonium hydroxide (TMAH) in methanol (hereafter referred to as TMAH). TMAH can liberate fatty acids bound in macromolecules or chemically bound monomers associated with mineral phases and make these organics detectable via gas chromatography mass spectrometry (GC-MS) by methylation. Fatty acids, a type of carboxylic acid that contains a carboxyl functional group, are of particular interest given their presence in both biotic and abiotic materials. This work represents the first analyses of a suite of Mars-analog samples using the TMAH experiment under select SAM-like conditions. Samples analyzed include iron oxyhydroxides and iron oxyhydroxysulfates, a mixture of iron oxides/oxyhydroxides and clays, iron sulfide, siliceous sinter, carbonates, and shale. The TMAH experiments produced detectable signals under SAM-like pyrolysis conditions when organics were present either at high concentrations or in geologically modern systems. Although only a few analog samples exhibited a high abundance and variety of fatty acid methyl esters (FAMEs), FAMEs were detected in the majority of analog samples tested. When utilized, the TMAH thermochemolysis experiment on SAM could be an opportunity to detect organic molecules bound in macromolecules on Mars. The detection of a FAME profile is of great astrobiological interest, as it could provide information regarding the source of martian organic material detected by SAM.

## 1. Introduction

The ongoing exploration of Gale Crater by the NASA Mars Curiosity rover has modernized our understanding of martian geochemistry (Glavin *et al.,* 2013; McLennan *et al.,* 2014; Ming *et al.,* 2014; Anderson *et al.,* 2015; Freissinet *et al.,* 2015; Mangold *et al.,* 2016; Eigenbrode *et al.,* 2018), mineralogy (McAdam *et al.,* 2014; Vaniman *et al.,* 2014; Sutter *et al.,* 2017), sedimentology (Williams *et al.,* 2013; Edgar *et al.,* 2017; Mangold *et al.,* 2017), modern aeolian processes (Bridges *et al.,* 2017; Cousin *et al.,* 2017), planetary evolution (Atreya *et al.,* 2013; Mahaffy *et al.,* 2015), and potential for the presence of habitable environments (Grotzinger *et al.,* 2014). Ancient terrains on Mars continue to be sites of great astrobiological interest, and future missions such as the joint ESA–Roscosmos 2020 ExoMars rover and the NASA Mars2020 rover will be equipped to search for signs of past life on Mars (Mustard *et al.,* 2013). One of the promising techniques for life detection is wet chemistry pyrolysis gas chromatography mass spectrometry (py-GC-MS), which is available on the Curiosity rover as part of the Sample Analysis at Mars (SAM) instrument. Thermal volatilization gas chromatography mass spectrometry (TV-GC-MS) techniques limited to 500°C without wet chemistry capabilities have been utilized in the past by the Viking landers (Biemann *et al.,* 1977), and TV-MS and wet chemistry experiments limited to 500°C were utilized by the Phoenix lander (Boynton *et al.,* 2001). Py-GC-MS will again be used on the upcoming ExoMars rover as part of the Mars Organic Molecule Analyzer (MOMA) instrument (Goetz *et al.,* 2016; Goesmann *et al.,* 2017). The SAM suite configuration includes a tunable laser spectrometer (TLS), quadrupole mass spectrometer (QMS), gas chromatograph (GC), and the capability to perform wet chemistry (derivatization and thermochemolysis) analyses (Mahaffy *et al.,* 2012). The SAM Sample Manipulation System (SMS) carousel contains 74 individual sample cups housed within two concentric rings of the carousel. Nine of these cups are slated for wet chemistry experiments and contain reagents for either thermochemolysis (two cups with tetramethylammonium hydroxide [TMAH] in methanol) or derivatization (seven cups with *N*-methyl-*N-tert*-butyldimethylsilyl-trifluoroacetamide [MTBSTFA] in dimethylformamide [DMF], hereafter referred to together as MTBSTFA).

**FIG. 1.**
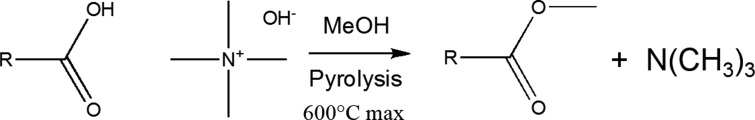
Schematic of the thermochemolysis reaction between a generic carboxylic acid (either a free fatty acid or membrane-bound fatty acid liberated from a macromolecule) and TMAH. Methanol is the solvent. The carboxylic acid is methylated at between 400°C and 600°C, and TMA is generated as a by-product.

Wet chemistry experiments aim to transform polar organic molecules (*e.g.,* carboxylic acids) into volatile derivatives that are amenable and detectable by gas chromatography mass spectrometry (GC-MS) (Metcalffe and Wang, 1981; del Rio *et al.,* 1996), as well as freeing bound components of larger macromolecules otherwise undetectable with GC-MS (Grasset *et al.,* 2002), if the macromolecule has hydrolysable units of appropriate size. Thermochemolysis, in this case thermal hydrolysis and methylation, with TMAH has been used for several decades in a variety of terrestrial fields of study, including soil sciences (Schulten, 1996; Chefetz *et al.,* 2000; Deport *et al.,* 2006), petroleum geochemistry (Larter and Horsfield, 1993), and sedimentology (Pulchan *et al.,* 1997; Guignard *et al.,* 2005; Remusat *et al.,* 2005; Anderson *et al.,* 2015). When strongly basic TMAH in methanol is added to a sample with membrane-bound phospholipids and heated, the phospholipids will undergo transesterification, liberating the fatty acids from their glycerol backbone. This creates a fatty acid carboxyl group which will be methylated to form a fatty acid methyl ester, or FAME (Fig. 1). FAMEs are more volatile than non-methylated fatty acids and therefore more amenable to detection by GC-MS.

Fatty acids are ubiquitous and abundant constituents of bacterial and eukaryotic cellular membranes, bound in phospholipids and glycolipids (Vestal and White, 1989). Carboxylic acids have also been identified in exogenous carbonaceous material, for example having been detected in the Murchison meteorite (Cronin *et al.,* 1993). In addition, lipids are common constituents of Mars analog environments on Earth (Parenteau *et al.,* 2014; Tan *et al.,* 2018). Biotic and abiotic carboxylic acids produce distinctly different GC-MS patterns. Fatty acids derived from microbial cellular metabolic processes have an even-over-odd carbon chain length preference due to enzymatically formed acetyl (C_2_) units derived from glucose (Volkman, 2006). In contrast, abiotic fatty acid patterns will favor shorter carbon chain lengths with no carbon preference (Bray and Evans, 1961; McCollom *et al.,* 1999). It is important to note that the even-over-odd carbon chain preference may be solely linked to terrestrial bacteria and may not reflect the carbon number chain preference of an extraterrestrial organism. In addition, very short chain carboxylic acids can be metabolic by-products that were not incorporated into cellular membranes. Therefore, with some level of accepted uncertainty, characteristics of a FAME profile could be used to deconvolve the origin of fatty acids detected on Mars (Eigenbrode *et al.,* 2011). FAME profiles have been used to explore microbial communities and preservation in terrestrial environments such as soils (Zelles, 1999; Ritchie *et al.,* 2000; Wilhelm *et al.,* 2017), acid mine drainage sediments (Ben-David *et al.,* 2004), carbonate nodules in freshwater stromatolites (Brady *et al.,* 2010), and shales (del Rio *et al.,* 1996; Blokker *et al.,* 2000).

**FIG. 2.**
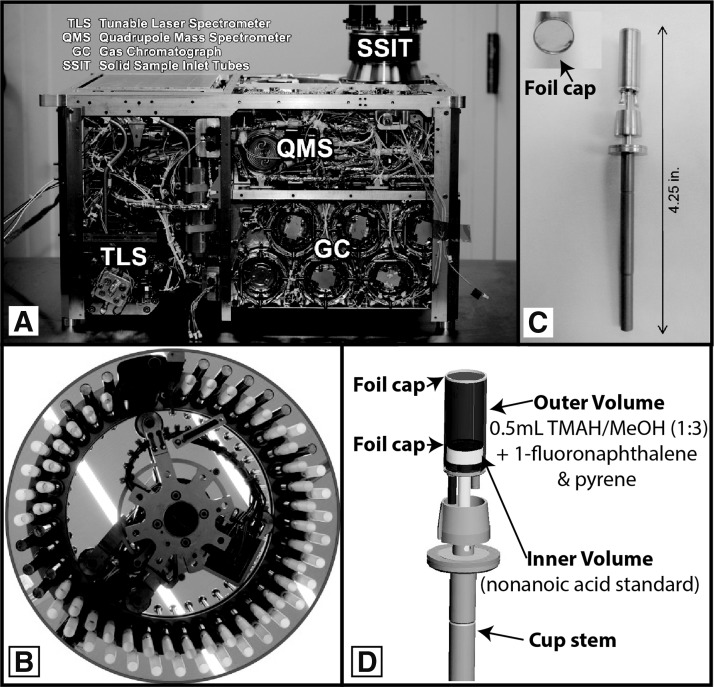
(**A**) The SAM instrument suite with side panels removed. (**B**) Examples of the foil-capped metal cups for wet chemistry experiments and the quartz cups for standard mass spectrometry analysis of solid samples. (**C**) A foil-capped wet chemistry cup (image of foil cap in inset). (**D**) Interior schematic of wet chemistry cup.

Figure 2 shows the SAM suite, SMS, and wet chemistry cups. Of the 74 sample cups housed within the SAM SMS carousel, two wet chemistry cups contain 0.5 mL total of TMAH in methanol (1:3 v/v), mixed with two recovery standards (1-fluoronaphthalene [34 nmol] and pyrene [25 nmol]), and nonanoic acid as an internal calibration standard. The nonanoic acid internal standard (*ca.* 12.5 nmol) is isolated from the TMAH inside a separate hermetically sealed internal foil-capped reservoir until the time of the thermochemolysis experiment in order to test the TMAH reaction efficiency. These cups were welded closed under N_2_ atmosphere to preserve the TMAH over the lifetime of the mission. As with the MTBSTFA experiment, pyrene is used as a recovery standard to ensure that the cup was fully punctured and the solvent was carried to the GC-MS. Additional details of the MTBSTFA experiment are presented elsewhere (Mahaffy *et al.,* 2012; Stalport *et al.,* 2012).

SAM carries six GC columns: four columns are dedicated to organics detection, and two are designed for very light hydrocarbon or small inorganic molecule analyses (Table 1). Of the organic-specific columns, GC1 (MXT-20) and GC5 (MXT-CLP) are both designed for medium molecular weight (C_5_–C_15_) organics, GC2 (MXT-5) is designed for high molecular weight (>C_15_) organics and derivative products, and GC4 (Chirasil-β-Dex CB) is designed to resolve enantiomers. When the TMAH experiment is conducted on Mars, the cup will be punctured with a pin located on the outer ring of the SMS. After puncture of the cup foils, the inner and outer reservoirs will mix to expose the nonanoic internal standard to the solvents, and the cup will rotate into position to receive the solid sample, composed of either the 1 mm or <150 μm size fraction of powdered drill fines, up to a volume of 790 μL. With solid sample delivery complete, the cup will be raised and sealed to the SAM pyrolysis oven. The cup will then be heated at 35°C min^−1^ from ambient to no higher than 600°C (the final temperature is still under development). Methylated thermochemolysis products will first be concentrated on the SAM hydrocarbon trap under He carrier gas flow at a rate of ∼0.03 atm-cc/s and then either concentrated on a GC injection trap that can be flash heated in order to quickly inject into the GC column (GC4 or GC5) or flow directly to a GC column (GC1 or GC2) at 0.9 mL min^−1^. The SAM hydrocarbon trap contains three adsorbents in the following order: 490 mg of 0.38 mm non-porous silica beads, 79 mg of 60/80 mesh Tenax TA, and 110 mg of 60/80 Carbosieve G (Mahaffy *et al.,* 2012). The gases will be trapped in the direction starting with the silica beads and ending with the Carbosieve G. The trap will then be heated and the gas flow reversed to release the trapped material. The adsorbed gases will be driven through one or more GC columns. The injection trap is composed of either Tenax GR (for GC4 and GC5) or Carbosieve III (for GC6). The methylated products are transferred to the cold trap, and the trap is flash heated to ∼310°C (over 5–10 s) to release the products to one of the GC columns designed for organics, in a sharp injection for a better GC separation. Once in the GC column of choice, the products are separated by using a column heating ramp up to 180–250°C. Methylated products will be identified based on their retention times and mass spectra as determined by the SAM QMS.

**Table 1.** Gas Chromatograph Columns on SAM

*Column*	*Species Targeted*
GC1 - MXT-20 (WCOT)	Medium molecular weight organics (C_5_–C_15_ organics)
GC2 - MXT-5 (WCOT)	High molecular weight VOCs including >C_15_ chemical derivatives
GC3 - Carbobond (PLOT)	Permanent gases and C_1_–C_2_ hydrocarbons
GC4 - Chirasil-β Dex CB	Enantiomers of VOCs
GC5 - MXT-CLP (WCOT)	Medium molecular weight organics (C_5_–C_15_ organics)
GC6 - MXT-Q (PLOT)	C_1_–C_4_ VOCs, NH_3_, S-containing compounds

WCOT = wall-coated open tubular, VOC = volatile organic compounds, PLOT = porous layer open tubes.

Here, we report the first GC-MS results of FAMEs produced from a selection of Mars-analog materials using a thermochemolysis procedure that approximates certain aspects of the SAM thermochemolysis wet chemistry experiment. Table 2 summarizes the main differences in conditions between the benchtop experimental approach used in this work and the SAM flight model. The goal of this study was to determine the recovery of FAMEs from a suite of rocks and sediments of various ages, including some samples considered mineralogic analogs to mineral suites observed on Mars. Utilizing Mars-analog samples will allow for contextualized results from the *in situ* experiment on Mars and provide valuable insight and testing of a wet chemistry technique slated for use on future flight instruments. Understanding the impact of mineralogical variations on thermal extraction and methylation is also of great importance (Ming *et al.,* 2009; Navarro-González *et al.,* 2010; Glavin *et al.,* 2013; Lewis *et al.,* 2015, 2018; Buch *et al.,* 2018). Fatty acids are the focus of this study (1) to provide a limited suite of organic compounds that may exhibit a large molecular weight range and (2) because TMAH thermochemolysis directly targets both free fatty acids and those bound in membranes. Results will reveal which mineral matrices have a positive or deleterious effect on the recovery of FAMEs through thermochemolysis experiments on a SAM-like benchtop system, defined in this work as using a SAM-like pyrolysis oven ramp of 35°C min^−1^ and SAM-comparable GC column and oven program with a commercial pyrolyzer and GC-MS. As a comparison to the SAM-like pyrolysis ramp, a 500°C flash pyrolysis method was used with the same procedure to determine how 500°C flash versus 35°C min^−1^ ramped pyrolysis affects FAME detection in the analog samples. Other “SAM-like” variables will be optimized in future work, such as He pressure, split, trap operation, and oven-trap gas processing system path length/temperature; therefore, those tests are outside the scope of this work. The work presented here will inform optimal sample selection in Gale Crater for employing this technique *in situ*.

**Table 2.** Comparison of Operating Conditions for Benchtop Experiments versus the SAM Flight Instrument

	*Benchtop py-GC-MS*	*SAM Flight Model*
Pyrolysis conditions	35°C min^−1^ or 500°C flash pyrolysis	35°C min^−1^
	800°C max stable oven temp	950–1100°C max oven temp depending on oven
Pyrolysis cup	Stainless steel cup	Stainless steel cup
Column Flow	3 mL min^−1^	∼0.9 mL min^−1^
Trap	In series: silica wool, Tenax TA, silica wool	In series: Silica beads, Tenax TA, Carbosieve G
Trap/Inlet Heater	300–310°C	>350°C
Transfer Line Temperature	135°C or 270°C	135°C
GC column	Restek MXT-CLP (GC5 comparable)	See column options in Table 1
GC conditions	Vary:	Vary:
	From ∼35°C to 300°C	From ∼10°C to max 160–260°C depending on column
	Ramp at 5°C min^−1^ or 10°C min^−1^ as desired	Ramp at 5°C min^−1^ or 10°C min^−1^ as desired

## 2. Materials and Methods

### 2.1. Analog samples and sample preparation

One cryoconite organic working sample, one procedural blank, and 13 terrestrial samples (Table 3) considered to have mineralogical relevance to martian environments of astrobiological interest were selected for this study and provide a variety of mineralogies that Curiosity could encounter in Gale Crater. Details on analog site sampling and sample preparation are in the Supplementary Materials (available at https://www.liebertpub.com/suppl/doi/10.1089/ast.2018.1819).

**Table 3.** Mineralogic Sample ID, Location, Age, and Dominant Mineral Species

*Group*	*Sample ID*	*Sample*	*Location*	*Age*	*Dominant Mineralogy*	*% TC ±0.060*	*% TOC ±0.060*
Organic Working Sample	CC	Cryoconite	Greenland Glacier	submodern	*Not determined*	nd	nd
Fused Silica	OCM	Organic Check Material	n/a	n/a	Fused silica quartz glass powder	nd	nd
Iron Oxides	PS5G	Surface gossan	Iron Mountain, CA	100s to 1000s of years old	Goethite	0.055	0.051
	SS12A	Pipeline precipitate	Iron Mountain, CA	Modern (months to years old)	Schwertmannite, goethite	0.86	0.97
	SSJ5	Acid saline lake sediment, from 1945 cm depth	Yilgarn Craton, Western Australia	Sub-Eocene	Quartz, halite, goethite, kaolinite, montmorillonite	0.074	0.073
	SSJ2	Acid saline lake sediment, from 2385 cm depth	Yilgarn Craton, Western Australia	Sub-Eocene	Quartz, halite, anatase, rutile, montmorillonite, ferruginous smectite	0.085	0.084
	SSJ3	Lake sediments; transition from circumneutral to acidic conditions, from 3905 cm depth	Yilgarn Craton, Western Australia	Sub-Eocene	Quartz, halite, goethite, maghemite, kaolinite, montmorillonite, illite, biotite, palygorskite	0.17	0.17
	SSJ4	Circumneutral lake sediment, from 4350 cm depth	Yilgarn Craton, Western Australia	Eocene	Quartz, halite, kaolinite, diopside, forsterite, montmorillonite	0.82	0.81
Iron Sulfide	PS5P	Surface gossan	Iron Mountain, CA	100s to 1000s of years old	Pyrite, quartz	0.056	0.029
Siliceous Sinter	IC160726.06.S	Inactive hot spring vent deposit (surface sample)	Gunnuhver, Iceland	Recent	Opal-CT	0.024	Assumed same as TC
	IC160726.06.I	Inactive hot spring vent deposit (7 cm deep sample)	Gunnuhver, Iceland	Recent	Opal-CT	0.011	Assumed same as TC
	IC160730.09.S	Modern hot spring vent deposit (surface sample)	Hveravellir, Iceland	Modern	Opal-A, clinopyroxenes (*e.g.,* augite, diopside), plagioclase, gypsum	0.24	Assumed same as TC
	IC160730.09.I	Modern hot spring vent deposit (4 cm deep sample)	Hveravellir, Iceland	Modern	Opal-A, plagioclase, diopside, magnesite	0.084	Assumed same as TC
Carbonates	CIMO	Cat Island modern ooid sand	Pigeon Bay, Cat Island, The Bahamas	Modern–Recent	Calcite	12	2.1^a^
Shale	MES	Messel Shale	Messel, Germany	Eocene	Clay minerals	33	33

^a^ Data from O'Reilly *et al.* (2017). TC = Total carbon determined by loss on ignition, TOC = Total organic carbon determined by HCl dissolution of carbonate and loss on ignition.

Total carbon and total organic carbon (TOC) were quantified by using a loss on ignition procedure on a Shimadzu TOC-Vcsh NC analyzer (detection limit 0.03–2.00 mg absolute, ±0.06% error). Sample mineralogy (Table 3) was determined on either a Bruker D8 Discover X-ray diffractometer or a Rigaku Ultima IV X-ray diffractometer. Samples were scanned from 2° to 64° 2θ. The measured patterns were then compared to standard mineral patterns from the RRuff repository (Downs, 2006) and the International Centre for Diffraction Data (ICDD) files with PDXL software as well as Materials Data Incorporated (MDI) Jade software to characterize the sample mineralogies.

Cryoconite was chosen as the organic working sample with a natural mixture of organic molecules for repeated analyses of py-GC-MS parameters. This cryoconite is a contemporary organic-rich aeolian dust deposit containing mixed living microbial communities with deceased biomass, anthropogenic soot, and micrometer-scale rock particles from Friedrichsbreen glacier in Svalbard.

A fused silica powder (FS120, HP Technical Ceramics, LTD, 250–125 μm) was heated to 550°C for 8 h in air to combust all organics, including plastic polymers, and was used as a procedural blank. It was processed in the same manner as the organic working sample and analog samples (Mahaffy *et al.,* 2012). The same material is carried on board Curiosity as the Organic Check Material (OCM) and can be used as a procedural blank to quantify any organic contamination from the sample handling system (Conrad *et al.,* 2012; Mahaffy *et al.,* 2012).

Select iron-rich samples were collected from the Iron Mountain massive sulfide deposit near Redding, California (Williams *et al.,* 2015). The massive sulfide surface exposure, also known as a gossan, consists predominantly of goethite with minor hematite and residual pyrite, and represents an acidic and saline system (Williams *et al.,* 2015; Jacobs *et al.,* 2016). The age of the modern surface gossan is not known and therefore is referred to here as “older,” for example, hundreds to thousands of years old.

Modern iron-rich precipitate was collected along an acid mine effluent pipeline at Iron Mountain. The pipeline carries pH 2.5–3.0 acid mine drainage water, and within the pipeline microbial oxidation of the iron-rich fluids creates an Fe(III)-rich schwertmannite mineral precipitate (Williams *et al.,* 2017).

Modern to Eocene-age mixed iron oxyhydroxides, halite, gypsum, quartz, and clay mineral bearing sediments were collected from an acid to circumneutral saline lake sediment core in the Norseman region, Yilgarn Craton, Western Australia. Sample SSJ5 was collected from 1945 cm depth, SSJ2 was collected from 2385 cm depth, SSJ3 was collected from 3905 cm depth, and SSJ4 was collected from 4350 cm depth. These iron-rich samples from the United States and Australia are considered to be mineralogically analogous to saline and circumneutral or acidic iron-dominated environments on Mars such as Meridiani Planum (Klingelhofer *et al.,* 2004; Benison and Bowen, 2006), and possibly the Vera Rubin Ridge (hematite-bearing unit) in Gale Crater (Fraeman *et al.,* 2013, 2016; Williams *et al.,* 2015).

Hot spring sinter deposits reported here are analogous to sinter deposits from deep-seated volcanically driven hydrothermal systems detected in Nili Patera, Mars (Skok, 2010). Actively forming sinter was collected from the Hveravellir hot spring, Iceland. A surface sample and 4 cm deep sample were collected from the modern lower vent. Sinter from an inactive but still hot spring mound was collected at the Gunnuhver hot springs, Iceland. A surface sample and 7 cm deep sample were collected from a lower vent sinter deposit.

Although extensive carbonate deposits have not been identified on Mars (Albee *et al.,* 2001), evidence for calcium carbonate has been found at the polar Phoenix landing site (Boynton *et al.,* 2009), and regionally limited deposits of hydrothermal magnesium-iron-carbonate were identified at Columbia Hills, Gusev Crater (Morris *et al.,* 2010; Carter and Poulet, 2012; Ruff *et al.,* 2014). Carbonates may also be present in other environments on Mars not yet explored *in situ* (Ehlmann *et al.,* 2008; Murchie *et al.,* 2009; Michalski and Niles, 2010). To serve as a carbonate mineral endmember, a modern oolitic sand was sampled from Pigeon Cay on Cat Island, The Bahamas. Ooids are grains 0.2–2 mm in diameter formed of concentrically or radially laminated mineral (commonly carbonate) layers surrounding a nucleus. Ooid cortices are known to contain and preserve organic matter (O'Reilly *et al.,* 2017).

Organic-rich shales are not expected on Mars; however, the Messel Shale from Messel, Germany, served to demonstrate the utility of the TMAH pyrolysis technique with an organic-rich endmember sample. The Messel Shale formed in an anoxic lacustrine environment in the Eocene and contains a great diversity and abundance of well-preserved organic molecules (Hayes *et al.,* 1987; Goth *et al.,* 1988).

### 2.2. Thermochemolysis reagent tetramethylammonium hydroxide

Wet chemistry experiments or derivatization techniques are capable of transforming polar organic molecules into more volatile forms that are detectable by GC-MS (Schummer *et al.,* 2009) and/or freeing bound components of larger macromolecules (Grasset *et al.,* 2002). In previous studies, TMAH thermochemolysis using pyrolysis is used to detect both free and membrane-bound fatty acids through methylation (Pulchan *et al.,* 1997; Chefetz *et al.,* 2000; Guignard *et al.,* 2005; Deport *et al.,* 2006), and transesterification of esters and methylation (Grasset *et al.,* 2002). Through this technique, a total FAME “profile” is obtained for a given sample.

Tetramethylammonium hydroxide (25%) in methanol (Sigma-Aldrich, P/N 334901, purity: <10% water, <2% chloride) is the thermochemolysis reagent used on SAM. During thermochemolysis, TMAH acts as the agent of transesterification, hydrolyzing and methylating fatty acids to generate a FAME that is more volatile and detectable by GC-MS. On SAM, *in situ* methylation with TMAH and a flash pyrolysis step <600°C will generate FAMEs from both membrane-bound fatty acids and free fatty acids, if present, such that the total FAME profile generated includes both fatty acid categories and cannot be discriminated between based on this technique.

### 2.3. Pyrolysis GC-MS conditions for analog samples

Aliquots of each powdered sample were carried through (1) a SAM-like 35°C min^−1^ pyrolysis ramp or (2) a 500°C flash pyrolysis step, then GC-MS analysis to measure the distribution and abundance of FAMEs. Ground rock or sediment samples were weighed into nonreactive metal cups (sample mass was *ca.* 5–10 mg, depending on the sample). Just prior to sample analysis, TMAH in the ratio of 1 mg sample to 1 μL TMAH was added to the cup. The *n*-C_19:0_ internal standard was injected into each sample immediately prior to analysis to determine the reaction efficiency. The sample was then immediately loaded and dropped into the commercial Frontier 3030D pyrolyzer oven to begin the run. The hydrocarbon trap consisted of silica wool, *ca.* 30 mg of Tenax TA, and a cap of silica wool within a glass inlet liner. The Tenax TA was initially conditioned at 320°C at a 1:40 split for 2 h. Gas is directed in one direction across the inlet liner, to trap and desorb the molecules of interest for GC-MS analysis. The Agilent 7890A-5975C inertXL GC-MS was equipped with a 30 m Restek capillary column (MXT-CLP) with a 0.25 mm internal diameter and 0.25 μm thick Crossbond 5% diphenyl/95% dimethyl polysiloxane stationary phase, with He as the carrier gas at a 3 mL min^−1^ flow rate and 10:1 split. Blank (without TMAH) and “TMAH-blank” (with TMAH) cleanup analyses were run between each sample to determine and subtract the background level of residual FAMEs in the column. The mass spectrometer scanned from amu 33–550 at a rate of 2.82 scans/s. The pyrolyzer and GC programs used for benchtop optimization of these experiments are detailed in the Supplementary Materials.

### 2.4. Duration of sample exposure to TMAH

Time trials were performed to determine (1) whether extended sample exposure to TMAH prior to pyrolysis would degrade FAME generation and detection, and (2) the amount of time at room temperature and ambient pressure that TMAH could interact with a sample and still yield detectable FAMEs. TMAH was added to aliquots of the cryoconite sample and pyrolysis and analysis of the samples was delayed to 1, 2, 6, 16, 25, 56, and 92 h after TMAH was introduced. One sample set was not delayed (0 h) and was run immediately after TMAH introduction. Delayed samples were stored under an organically clean watch glass under ambient atmosphere in a hood. Sample analysis utilized flash pyrolysis at 500°C and the GC-MS program described in the Supplemental Material.

### 2.5. TMAH/MeOH reactions with MTBSTFA/DMF vapor

MTBSTFA vapor is a known component within the SAM SMS, due to one or more MTBSTFA cup leaks during or after entry, descent, and landing on Mars (Glavin *et al.* 2013; Freissinet *et al.,* 2015). Therefore, the effect that MTBSTFA vapor may have on TMAH thermochemolysis experiments was tested. A mixture of 0.2 μL MTBSTFA in 500 μL TMAH (to approximate the low concentration of MTBSTFA vapor that adsorbs to the cups in the SMS) was introduced into the py-GC-MS system with the goethite sample PS5G to identify any pyrolysis by-products.

### 2.6. TMAH concentration and loss of methanol before sample pyrolysis

In order to determine how the concentration of TMAH may be affected by the evaporation of MeOH from the mixture, experiments were conducted to determine the evaporation rate curve for MeOH at Mars-like pressures and temperatures (down to 3°C), as well as SAM SMS-like temperatures (up to 50°C). Aliquots (*ca.* 0.5 g) of TMAH in MeOH were weighed out and held within a Mars-like pressure range (0.93–1.33 kPa) at 3°C, 27°C, 40°C, 45°C, and 50°C. A similar experiment was conducted at Mars pressures using MTBSTFA in DMF at 45°C. The remaining masses were measured at 0, 10, 15, 20, 25, 35, and 45 min, and assuming methanol-only evaporation (as methanol evaporates at 64.6°C whereas TMAH only decomposes), the amount and concentration of TMAH remaining was calculated.

### 2.7. Sample peak identification

Chromatograms and the mass spectra for FAMEs were analyzed with ChemStation software (Agilent Technologies). Identifications were based on comparison to known FAME retention times of a Supelco 37 component FAME mixture that contains saturated FAMEs, monounsaturated fatty acid methyl esters (MUFAMEs), and polyunsaturated fatty acid methyl esters (PUFAMEs) (Sigma-Aldrich). In addition, mass spectra were compared to the National Institute of Standards and Technology (NIST) Spectral Library. Quantifications were based on integration under peaks and scaled to the known amount of the *n-*C_19:0_ internal standard unless otherwise noted.

## 3. Results

### 3.1. Duration of sample exposure to TMAH

**FIG. 3.**
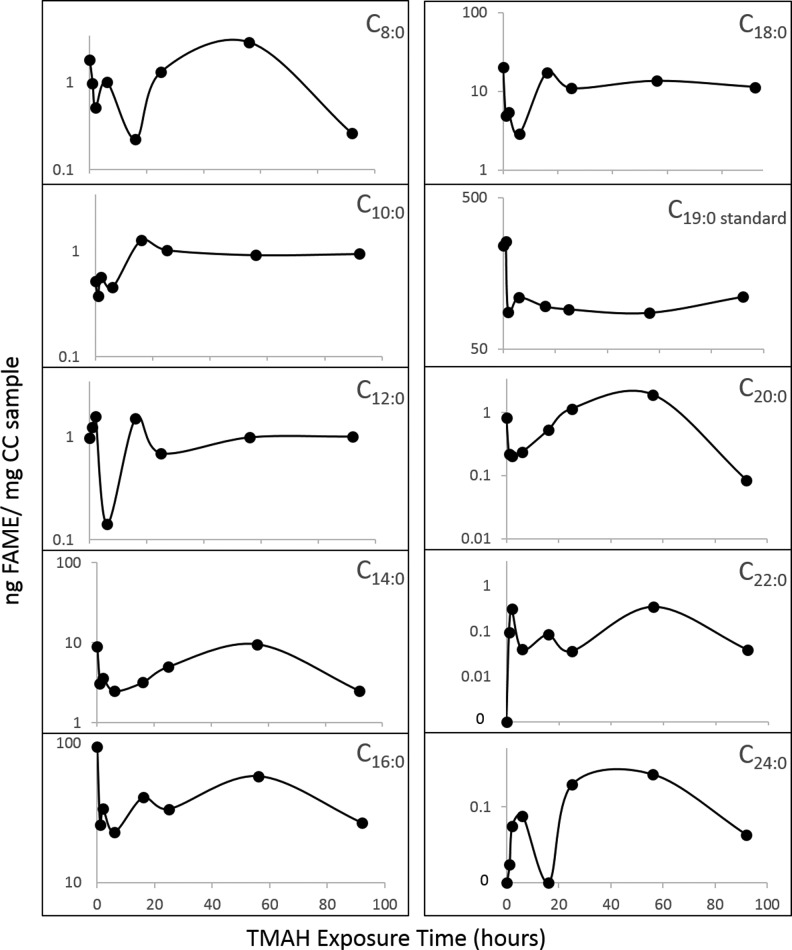
Time tests for cryoconite exposure to TMAH, including instant (0 h delay), 1, 3, 6, 16, 25, 56, and 92 h delay before pyrolysis, for C_8:0_ to C_24:0_. Note the different *y*-axis scales. FAME concentrations were in general highest in the 0 h trial, but FAMEs were detectable up to 92 h after TMAH exposure and prior to pyrolysis.

The concentration of select FAMEs for the eight time trials is shown in Fig. 3. The C_19:0_ internal standard concentration was highest in the 0 and 1 h trials but never dropped below 86 ng/mg cryoconite (CC) (66% of maximum). The more abundant FAMEs, C_14:0_, C_16:0_, and C_18:0_, were highest in the 0 h trials and never dropped below 2.5, 23, and 2.9 ng/mg CC (74%, 76%, and 86% of maximum, respectively). The less abundant FAMEs (C_8:0_, C_10:0_, C_12:0_, C_20:0_, C_22:0_, and C_24:0_) had concentrations around 0.1–1 ng/mg CC, putting them within the range of detection, albeit at low concentrations. Of note, the lower molecular weight FAMEs that are most likely to be detected in the SAM experiment yielded sufficiently elevated concentrations to be detected even after 92 h of exposure. Only C_22:0_ was not detected at 0 h exposure, and C_24:0_ at 0 and 16 h exposure.

### 3.2. TMAH/MeOH reactions with MTBSTFA/DMF vapor

**FIG. 4.**
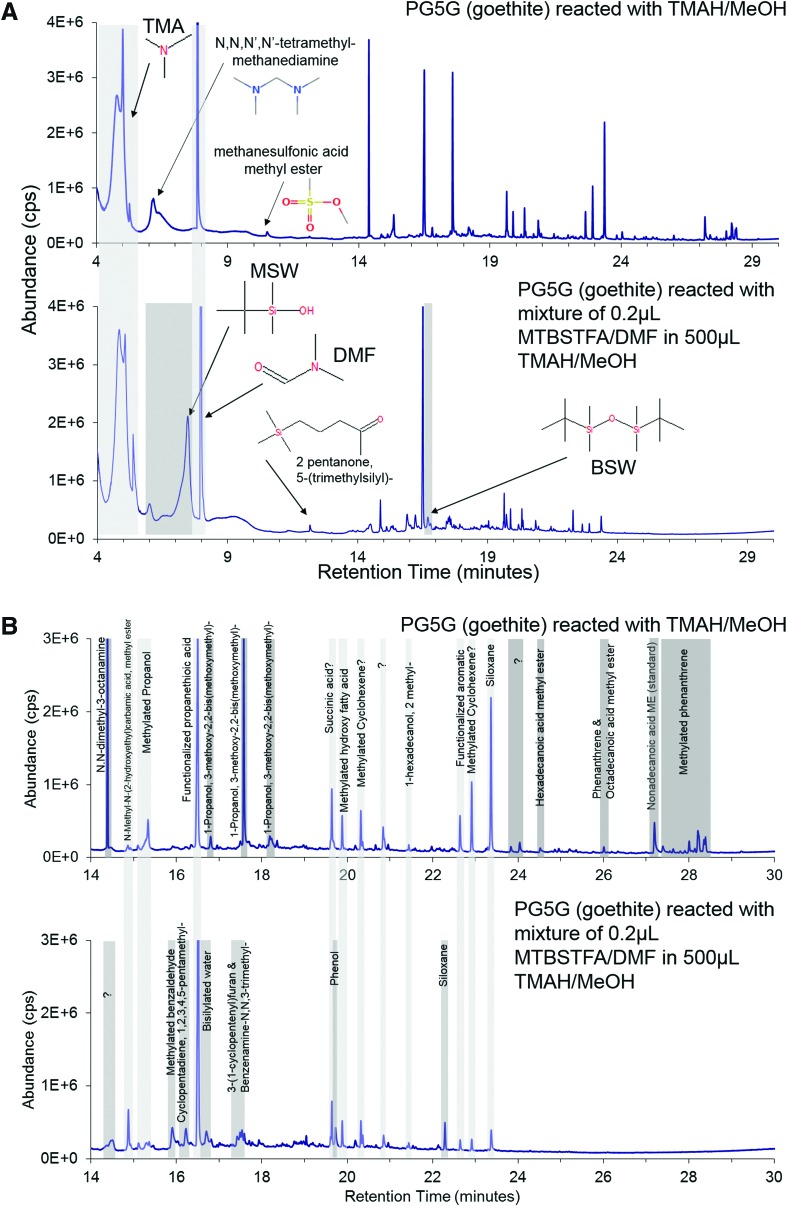
(**A**) Total ion chromatogram of goethite sample reacted with TMAH/MeOH compared to goethite sample reacted with mixture of 0.2 μL MTBSTFA/DMF in 500 μL TMAH/MeOH. TMA is shared between the two tests, whereas the MTBSTFA by-products DMF, monosilylated water (MSW), and bisilylated water (BSW) are present in the mixed reagent. The DMF peak in the goethite reacted with TMAH is residual from a previous analysis. The siloxanes are a common product of column bleed. No internal standard was included in this analysis. (**B**) Total ion chromatogram enhancement of R_t_ = 14–30 min from (A) showing methylated and/or silylated products from the goethite sample reacted with TMAH/MeOH compared to the goethite sample reacted with mixture of 0.2 μL MTBSTFA/DMF in 500 μL TMAH/MeOH. Many of the same methylated compounds are detected in both experiments, with additional compounds detected separately in either experiment. Color images are available online at www.liebertpub.com/ast.

The goethite sample PS5G was exposed to (1) TMAH and (2) the 0.2 μL MTBSTFA in 500 μL TMAH mixture. In the goethite reacted with TMAH test, the known TMAH by-product trimethylamine (TMA) was generated, and the MTBSTFA by-product DMF was present as a carryover from the previous analysis containing MTBSTFA (Fig. 4A). The double TMA peak is likely an artifact of partial TMA solvation by MeOH. In the goethite reacted with MTBSTFA in TMAH test, TMA and DMF were generated, as were the MTBSTFA by-products *tert*-butyldimethylsilanol (monosilylated water or MSW, *ca.* 5.9 and 7.4 min) and 1,3-bis(1,1-dimethylethyl)-1,1,3,3-tetramethyldisiloxane (bisilylated water or BSW, *ca.* 16.7 min, Fig. 4A). The MTBSTFA by-product *N*-methyl-2,2,2-trifluoroacetamide (TFMA) was not detected. As this experiment was performed with a natural goethite sample, a variety of methylated and silylated products were generated in both tests (Fig. 4B). The goethite reacted with TMAH test also generated products not shared with the mixed reagent test, for example, methylated phenanthrene and FAMEs. The goethite reacted with MTBSTFA in TMAH test generated products unique to that experiment, for example, methylated benzaldehyde, 1,2,3,4,5-pentramethyl-cyclopentadiene, 3-(1-cyclopentenyl)furan, N,N,3-trimethyl-benzenamine, phenol, and siloxanes.

### 3.3. TMAH concentration and loss of methanol prior to sample pyrolysis

**FIG. 5.**
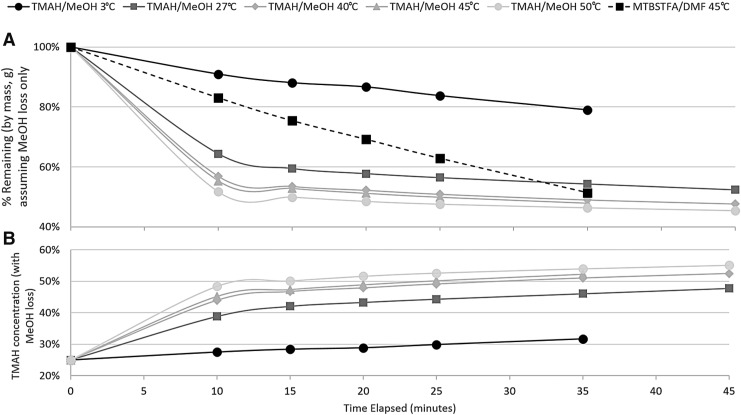
(**A**) Percent of mass remaining after MeOH evaporation from TMAH/MeOH mixture at Mars-like pressure (0.93–1.33 kPa) and various temperatures: Mars-like: 3°C; SAM SMS-like: 27°C, 40°C, 45°C, 50°C. For comparison, the evaporation curve for MTBSTFA in DMF at 45°C is reported. (**B**) Percent concentration of TMAH in TMAH/MeOH mixture with MeOH evaporation at Mars-like pressure (0.93–1.33 kPa) and various temperatures: Mars-like: 3°C; SAM SMS-like: 27°C, 40°C, 45°C, 50°C.

Experiments were conducted to determine the evaporation rate for MeOH from the TMAH in MeOH mixture at Mars-like pressures and temperatures. Results demonstrate that within 10 min, between 9% (at 3°C) and 48% (at 50°C) of the MeOH in the original TMAH/MeOH mixture evaporates (Fig. 5). This changes the concentration of TMAH in the mixture after 10 min from 25% to 27% (at 3°C) and 25% to 48% (at 50°C) (Fig. 5). Results indicate that the loss of MeOH from the 25% TMAH in MeOH mixture will be minimized at the low operating temperatures on Mars and should not significantly affect the *in situ* experiment based on similar benchtop experiments of MeOH evaporation from TMAH/MeOH at room temperature and ambient pressure.

### 3.4. Thermochemolysis and pyrolysis GC-MS analysis of analog samples

**FIG. 6.**
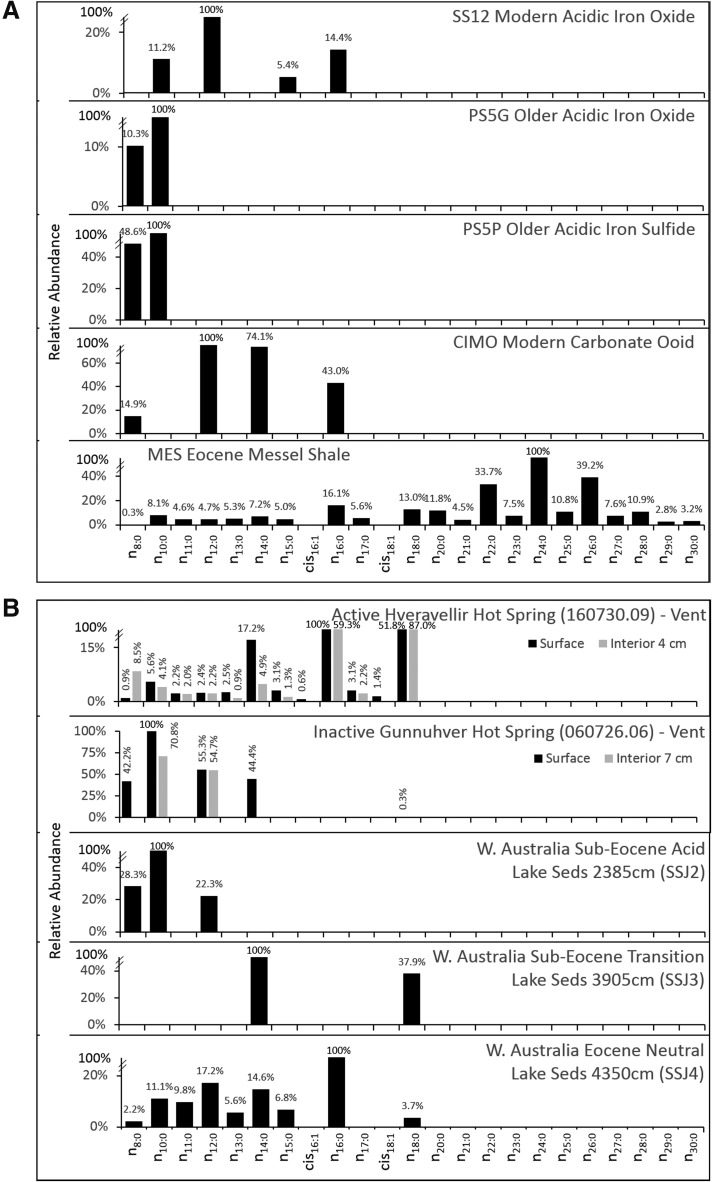
(**A**) Percent relative abundance of FAME profiles for the mineral classes analyzed with a SAM-like 35°C min^−1^ pyrolysis ramp. The modern acidic iron oxide was dominated by *n*-C_10:0_, *n*-C_12:0_, *n*-C_15:0_, and *n*-C_16:0_ FAMEs. The older iron oxide and iron sulfides contained only *n*-C_8:0_ and *n*-C_10:0_ FAMEs. The modern carbonate ooid was dominated by *n*-C_12:0_, *n*-C_14:0_, and *n*-C_16:0_ FAMEs. The Eocene Messel Shale contained a great diversity of FAMEs and was dominated by *n*-C_24:0_ and other long chain length FAMEs up to *n*-C_30:0_. As the C_19:0_ internal standard was not detected in all ramped pyrolysis analyses, abundance in each profile is normalized to the highest FAME cps detection from that sample. (**B**) Percent relative abundance of FAME profiles for the mineral classes analyzed with a SAM-like 35°C min^−1^ pyrolysis ramp (continued). The active Icelandic hot spring was dominated by *n*-C_16:0_ and *n*-C_18:0_ FAMEs in the surface and interior samples and contained several shorter-length FAMEs. The recently active hot spring was dominated by *n*-C_10:0_ in the surface and interior samples. The acidic sub-Eocene lake sediment (SSJ2) was dominated by *n*-C_10:0_, and the circumneutral Eocene lake sediment (SSJ4) was dominated by *n*-C_16:0_. As the C_19:0_ internal standard was not detected in all ramped pyrolysis analyses, abundance in each profile is normalized to the highest FAME cps detection from that sample.

**FIG. 7.**
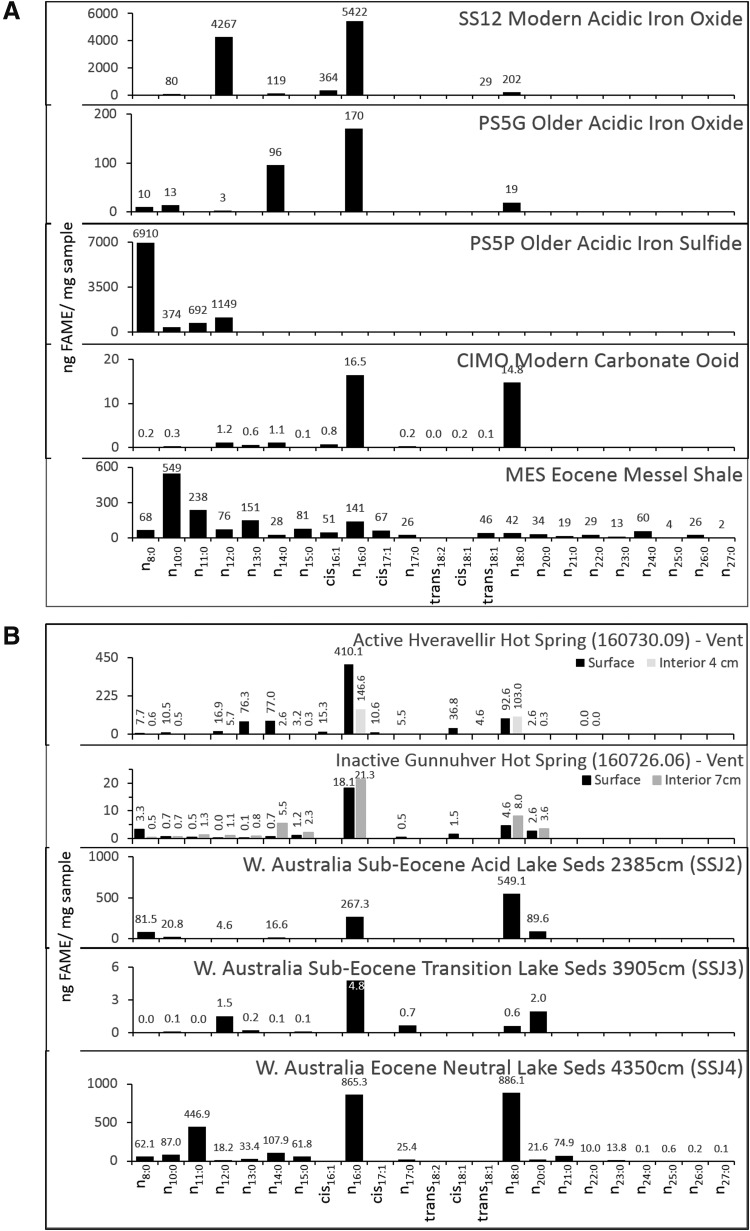
(**A**) FAME profiles for the mineral classes analyzed with the 500°C flash pyrolysis method. FAMEs are quantified as ng FAME/mg sample. Most samples are dominated by *n*-C_16:0_ and *n*-C_18:0_. The SS12 sample contains a modern microbial community and had a very high concentration of FAMEs/mg sample relative to older samples or those in which the microbial communities had been entombed by minerals. The high FAME concentration in the PS5P sample indicates a modern microbial community within the older sample. The even-over-odd carbon number preference is apparent in the FAMEs > *n*-C_18:0_ from the Messel Shale. (**B**) FAME profiles for the mineral classes analyzed with the 500°C flash pyrolysis method (continued). Most samples are dominated by *n*-C_16:0_ and *n*-C_18:0_. The active Icelandic hot spring contained a higher FAME concentration than the inactive hot spring system. An odd-over-even carbon number preference is observed in the FAMEs > *n*-C_18:0_ from the Western Australia neutral lake sediments, which also contain the highest concentration of FAMEs of the Western Australia samples.

The generation of FAMEs from a suite of Mars analog materials was tested by using both a SAM-like pyrolysis ramp and a 500°C flash pyrolysis (Table 3). Results are shown in Table 4, Supplementary Table S1, and Figs. 6 and 7. The *n-*C_19:0_ internal standard was detected in all samples except those noted below.

**Table 4.** Presence or Absence of FAMEs in Mars-Analog Samples Analyzed with Either a SAM-like 35°C min^−1^ Pyrolysis Ramp or a 500°C Flash Pyrolysis Step

	*SS12 modern iron precipitate*	*PS5G older iron oxides*	*PS5P older pyrite*	*CIMO modern ooids*	*MES*	*SSJ2*	*SSJ3*	*SSJ4*	*SSJ5*	*160726.06.S Gunnuhver vent, recent*	*160726.06.I Gunnuhver vent, recent*	*160730.09.S Hveravellir vent, active*	*160730.09.I Hveravellir vent, active*
*SAM-like 35°C min^−1^ pyrolysis ramp*													
C4	–	–	–	–	–	X	–	–	–	–	–	–	–
C6	–	X	X	X	X	X	–	X	–	–	X	X	–
C8	–	X	X	X	X	X	–	X	–	X	–	X	X
C9	–	X	X	–	X	X	–	X	–	X	X	X	X
C10	X	X	X	–	X	X	–	X	–	X	X	X	X
C11	–	–	–	–	X	–	–	X	–	–	–	X	X
C12	X	–	–	X	X	X	–	X	–	X	X	X	X
C13	–	–	–	–	X	–	–	X	–	–	–	X	X
C14	–	–	–	X	X	–	X	X	–	X	–	X	X
C15	X	–	–	–	X	–	–	X	–	–	–	X	X
C16	X	–	–	X	X	–	–	X	–	–	–	X	X
C16:1	–	–	–	–	–	–	–	–	–	–	–	X	–
C17	–	–	–	–	X	–	–	–	–	–	–	X	X
C18	–	–	–	–	X	–	X	X	–	X	–	X	X
C18:1n9c	–	–	–	–	–	–	–	–	–	–	–	X	–
C18:2	–	–	–	–	–	–	–	–	–	–	–	–	–
*C19 STD*	–	–	–	X	X	–	–	–	–	X	X	X	X
C20	–	–	–	–	X	–	–	–	–	–	–	–	–
C21	–	–	–	–	X	–	–	–	–	–	–	–	–
C22	–	–	–	–	X	–	–	–	–	–	–	–	–
C23	–	–	–	–	X	–	–	–	–	–	–	–	–
C24	–	–	–	–	X	–	–	–	–	–	–	–	–
C25	–	–	–	–	X	–	–	–	–	–	–	–	–
C26	–	–	–	–	X	–	–	–	–	–	–	–	–
C27	–	–	–	–	X	–	–	–	–	–	–	–	–
C28	–	–	–	–	X	–	–	–	–	–	–	–	–
C29	–	–	–	–	X	–	–	–	–	–	–	–	–
C30	–	–	–	–	X	–	–	–	–	–	–	–	–
*500°C Flash Pyrolysis*													
C4	–	–	–	–	X	–	–	X	–	–	–	–	–
C5	–	–	–	–	X	–	–	–	–	–	–	–	–
C6	–	–	X	–	X	–	–	X	X	X	X	X	–
C7	–	–	–	–	X	–	–	–	–	–	–	–	–
C8	–	X	X	X	X	X	X	X	X	X	X	X	X
C9	X	X	X	X	X	X	X	X	X	X	X	X	X
C10	X	X	X	X	X	X	X	X	X	X	X	X	X
C11	–	–	X	–	X	–	X	X	X	X	X	–	–
C12	X	X	X	X	X	X	X	X	X	X	X	X	X
C13	–	–	–	X	X	–	X	X	X	X	X	X	–
C14	X	X	–	X	X	X	X	X	X	X	X	X	X
C15	–	–	–	X	X	–	X	X	X	X	X	X	X
C16:1	X	–	–	X	X	–	–	–	–	–	–	X	–
C16	X	X	–	X	X	X	X	X	X	X	X	X	X
C17:1	–	–	–	–	X	–	–	–	–	–	–	X	–
C17	–	–	–	X	X	–	X	X	X	X	–	X	–
C18:2	–	–	–	X	–	–	–	–	–	–	–	–	–
C18:1n9t	–	–	–	X	X	–	–	–	–	–	–	X	–
C18:1n9c	X	–	–	X	–	–	–	–	–	X	–	X	–
C18	X	X	–	X	X	X	X	X	X	X	X	X	X
*C19 STD*	X	X	X	X	X	X	X	X	X	X	X	X	X
C20	–	–	–	–	X	X	X	X	–	X	X	X	X
C21	–	–	–	–	X	–	–	X	–	–	–	–	–
C22	–	–	–	–	X	–	–	X	–	–	–	X	X
C23	–	–	–	–	X	–	–	X	–	–	–	–	–
C24	–	–	–	–	X	–	–	X	–	–	–	–	–
C25	–	–	–	–	X	–	–	X	–	–	–	–	–
C26	–	–	–	–	X	–	–	X	–	–	–	–	–
C27	–	–	–	–	X	–	–	X	–	–	–	–	–

X = presence, – = absence.

#### 3.4.1. Fused silica (SAM Organic Check Material analog)

Small FAME peaks were detected in the fused silica sample by using both the background-subtracted SAM-like method (with 2 FAMEs—*n-*C_8:0_ and *n-*C_9:0_—identified) and the background-subtracted 500°C flash pyrolysis method (with 5 FAMEs identified: *n-*C_8:0_, *n-*C_10:0_, *n-*C_12:0_, *n-*C_16:0_, and *n-*C_18:0_). The effect of the background-subtraction approach is detailed in the discussion section, and all analog samples reported below represent background-subtracted analyses.

#### 3.4.2. Cryoconite organic working sample

**FIG. 8.**
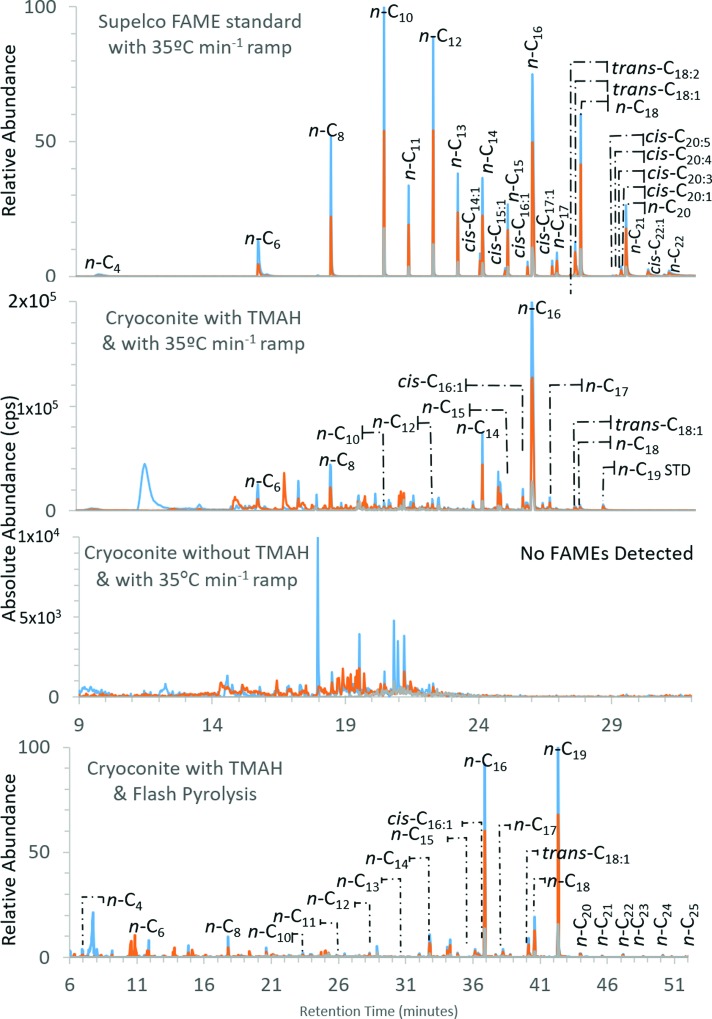
Selected ion chromatogram of Supelco FAME 37 standard and cryoconite analyzed by py-GC-MS. The FAME standard was used to confirm FAME retention times in the cryoconite sample treated with and without TMAH. The sample not reacted with TMAH did not yield detectable FAMEs but did contain molecules such as trimethylsilyl cyanide, furfural, methylated furancarboxaldehyde, and siloxanes. The TMAH-reacted cryoconite analyzed by flash pyrolysis is included to demonstrate that flash pyrolysis yields more FAMEs than the ramped pyrolysis method. The 35°C min^−1^ and 500°C flash pyrolysis methods and GC programs are described in the Supplementary Materials. FAMEs identified by retention time and *m/z* = 74, 87, 143. Blue lines = *m/z* 74, orange lines = *m/z* 87, gray lines = *m/z* 143. Color images are available online.

Eleven FAMEs and MUFAMEs were detected in the cryoconite sample treated with TMAH, while no FAMEs were detected in the cryoconite sample that did not undergo thermochemolysis with TMAH (Fig. 8). With the 500°C flash pyrolysis method, which used TMAH, 21 FAMEs and MUFAMEs ranging from *n*-C_4:0_ to *n*-C_25:0_ were detected.

#### 3.4.3. Iron Mountain, California, iron oxyhydroxides and iron oxyhydroxysulfates

The Iron Mountain gossan sample (PS5G, 0.051% TOC) was dominated by goethite and yielded four FAMEs below *n*-C_11:0_ with the SAM-like method, and the internal standard was not detected. The flash pyrolysis method generated 7 FAMEs below *n*-C_20:0_.

The modern Iron Mountain schwertmannite precipitate (SS12, 0.97% TOC) yielded 4 FAMEs below *n-*C_17:0_ with the SAM-like method, and the internal standard was again not detected. In contrast, the flash pyrolysis method generated 9 FAMEs or MUFAMEs with chain lengths shorter than *n*-C_20:0_.

#### 3.4.4. Western Australia mixed iron oxides/oxyhydroxides and clay minerals

The Western Australia acidic lake core sample SSJ5 (1945 cm depth, 0.073% TOC), which contains halite, goethite, and clay minerals, did not yield any FAMEs with the SAM-like method, and the internal standard was not detected. However, with the flash pyrolysis method, 12 FAMEs were detected below *n*-C_20:0_.

The acidic lake sediment core sample SSJ2 (2385 cm depth, 0.084% TOC) does not contain an iron oxide/oxyhydroxide mineral phase but does contain halite and clay minerals, including ferruginous smectite. With the SAM-like method, 5 FAMEs below *n*-C_13:0_ were detected, and the internal standard was not detected, whereas with the flash pyrolysis method 7 FAMEs below *n*-C_20:0_ were detected.

Lake sediment core sample SSJ3 (3905 cm depth, 0.17% TOC) represents the transition from more modern acidic and saline conditions to circumneutral lake sediments of Eocene age (Bowen and Benison, 2009; Benison and Bowen, 2015) and contains halite, goethite, maghemite, and the clay minerals kaolinite, montmorillonite, and illite. The SAM-like method only yielded *n*-C_14:0_ and *n*-C_18:0_ FAMEs, and the internal standard was not detected. The flash pyrolysis method yielded 11 FAMEs.

Eocene-age circumneutral lake sediment core sample SSJ4 (4350 cm depth, 0.81% TOC) contains, along with other mineral phases, halite, montmorillonite, and kaolinite. With the SAM-like method, 11 FAMEs below *n-*C_19:0_ were detected, and the internal standard was not detected, whereas 20 FAMEs below *n*-C_28:0_ were detected with the flash pyrolysis method.

#### 3.4.5. Iron Mountain, California, iron sulfide

The Iron Mountain pyrite sample (PS5P, 0.029% TOC), dominated by pyrite and quartz, yielded 4 FAMEs with the SAM-like method and 4 FAMEs with the flash pyrolysis method. The *n-*C_19:0_ internal standard was not detected using the SAM-like ramp.

#### 3.4.6. Icelandic siliceous sinter

The inactive near-vent sinter deposit from Gunnuhver, Iceland, demonstrated a decrease in FAME detection at depth with the SAM-like method. The surface sample (IC160726.06.S, 0.024% TOC) yielded 6 FAMEs. The subsurface sample (IC160726.06.I, 0.011% TOC) was collected at 7 cm depth and yielded 4 FAMEs. The flash pyrolysis method produced a similar trend, with 13 FAMEs and MUFAMEs detected in the surface sample and 11 FAMEs detected in the subsurface sample.

An active hot spring sinter deposit from Hveravellir, Iceland, demonstrated a similar decrease in FAME detection at depth with the SAM-like method. The surface sample (IC160730.09.S, 0.24% TOC) yielded 14 FAMEs. The subsurface sample (IC160730.09.I, 0.084% TOC) was collected at 4 cm depth and yielded 11 FAMEs. The flash pyrolysis method produced a similar trend, with 17 FAMEs, MUFAMEs, and PUFAMEs detected in the surface sample and 10 FAMEs detected in the subsurface sample.

#### 3.4.7. Bahamian carbonates

The modern carbonate ooid sample (CIMO, *ca.* 2.1% TOC) yielded 5 FAMEs with the SAM-like method and 17 FAMEs with the flash pyrolysis method.

#### 3.4.8. Organic-rich shale

Using the SAM-like method, the Messel shale sample (MES, 33% TOC) yielded 23 FAMEs and MUFAMEs, and with the flash pyrolysis method 42 FAMEs and MUFAMEs were detected.

## 4. Discussion

### 4.1. Benchtop and SAM flight thermochemolysis optimization

To optimize the TMAH thermochemolysis benchtop experiment, several operational variables were evaluated, including duration of sample exposure to TMAH, TMAH reactions with MTBSTFA, and evaporation of methanol from the TMAH solution. The sample exposure to TMAH tests suggest that the sample should be run as soon as possible after exposure to TMAH, but the sample integrity should not be compromised if the pyrolysis and analysis are delayed up to 92 h after sample exposure to TMAH (Fig. 3). The presence of MTBSTFA vapor will not significantly affect the TMAH experiment and expected methylated products. The loss of methanol from the TMAH/MeOH mixture at Mars temperatures will not significantly impact the experiment. The operating parameters described above will continue to be optimized for the SAM flight instrument. Further investigation will be conducted to optimize the flight experiment, including determining the exact percentage of sample-reacted TMAH to be vented from SAM versus sent to the GC-MS. Continued testing of the TMAH thermochemolysis experiment under Mars temperature and pressure conditions is planned for the SAM testbed, a high-fidelity replica of the SAM flight instrument housed at NASA Goddard Space Flight Center.

### 4.2. Thermochemolysis of analog samples

FAME generation via TMAH thermochemolysis in mineralogically variable Mars-analog samples was tested with both a SAM-like (35°C min^−1^) pyrolysis step and a 500°C flash pyrolysis step. In general, the flash pyrolysis method yielded a greater number and diversity of FAMEs from all samples than the SAM-like pyrolysis ramp method (Table 4). Common sources for the FAMEs identified are presented in Table 5 and interpreted below in the context of the sample environment. The FAME profiles generated with SAM-like pyrolysis are shown in Fig. 6, and those generated with flash pyrolysis are shown in Fig. 7, to demonstrate the distinct profile “fingerprints” of each mineralogy class.

**Table 5.** Fatty Acid Biomarkers Detectable with TMAH Thermochemolysis, Abbreviations, and Possible Biogenic Sources of Fatty Acids (Modified from O'Reilly *et al.,* 2017)

*Name*	*Abbreviation*	*Source and Interpretation*
Straight-chain saturated fatty acids (<20 carbons)	C_14:0_ to C_18:0_	Bacteria, eukaryotes^a^
Long-straight-chain saturated fatty acids	C_20:0_ to C_30:0_	Microalgae or terrestrial plants,^b,c^
		Early diagenetic selective preservation^d^
Hexadec-9-enoic acid	C_16:1ω7_	Diatoms, proteobacteria^e^
Hexadec-11-enoic acid	C_16:1ω5_	Bacteria, possibly sulfate-reducing bacteria^f^
Octadec-11-enoic acid	C_18:1ω7_	Bacteria, microalgae^e^
Octadec-9-enoic acid	C_18:1ω9_	Eukaryotes^a^
Octadecadienoic acid	C_18:2ω6_	Possibly cyanobacteria, fungi^g,h^
Loss of unsaturated fatty acids		Bacterial heterotrophy/early diagenesis^h^

^a^ Volkman, 2006; ^b^Volkman *et al.,* 1989; ^c^Wilhelm *et al.,* 2017; ^d^Haddad *et al.,* 1992; ^e^Volkman *et al.,* 1998; ^f^Elvert *et al.,* 2003; ^g^Kelly and Scheibling, 2011; ^h^Cantrell *et al.,* 2006.

#### 4.2.1. Fused silica (Curiosity rover Organic Check Material analog)

Given that this sample was combusted at 550°C for 8 h prior to analysis, and several TMAH-blanks were run before sample analysis, carryover contamination from previously run samples is the likely source of the very small FAME peaks detected. Evidence for this is discussed in detail in Section 4.2.10.

#### 4.2.2. Cryoconite organic working sample

The results from the SAM-like pyrolysis of the cryoconite sample with and without TMAH, and the 500°C flash pyrolysis with TMAH experiment, are shown in Fig. 8 and compared to the Supelco 37 FAME standard. These results are consistent with the expectation that TMAH will generate methylated fatty acids from ester-linked lipids, whereas samples not treated with TMAH will not yield FAMEs. The 500°C flash pyrolysis experiments yielded more FAMEs than the SAM-like ramp; this is described in more detail in Section 4.2.11.

#### 4.2.3. Iron Mountain iron oxyhydroxides and iron oxyhydroxysulfates

The straight-chain saturated FAMEs detected in the modern Iron Mountain precipitate (SS12) may be indicative of both bacterial and eukaryotic cell membranes (Volkman, 2006). The *cis*-C_16:1_ may indicate the presence of diatoms (which were identified via SEM in this sample) or proteobacteria (Volkman *et al.,* 1998), or possibly sulfate-reducing bacteria (Elvert *et al.,* 2003), although sulfate-reducing bacteria have not yet been identified in the modern pipe precipitate. The *cis*-C_18:1_ can indicate the presence of bacteria or microalgae (Volkman *et al.,* 1998) or eukaryotes (Volkman, 2006), depending on the location of the unsaturation.

Assuming unsaturated FAMEs were initially present in the older Iron Mountain gossan sample (PS5G) as they are in the modern sample, loss of those unsaturated FAMEs can indicate the effects of diagenesis or bacterial heterotrophy (Sun *et al.,* 1997). The low FAME detection in this sample may be due to a decreased initial microbial population within the gossan rock or a reduction in FAME preservation due to the presence of iron oxides/oxyhydroxides (Sumner, 2004).

#### 4.2.4. Western Australia mixed iron oxides/oxyhydroxides and clays

The Western Australia lake sediment core samples ranged from acidic at 1945 cm depth to circumneutral at 4350 cm depth. Several of these samples contained a mixture of iron oxide/oxyhydroxide and clay minerals, but environmental pH demonstrated the strongest influence on the preservation of fatty acids in these sediments. With both py-GC-MS methods, fewer FAMEs and lower FAME concentrations were detected in the acidic sediment samples relative to the circumneutral sediment samples, confirming the thermodynamic assumption that organics are more rapidly degraded in acidic rather than circumneutral environments. However, the resilience of the remaining FAMEs in these Eocene to sub-Eocene age samples suggests that the mixture of iron oxides/oxyhydroxides and clay minerals preserves organics better than iron oxides/oxyhydroxides alone (*e.g.* in the PS5G goethite sample).

The long straight-chain saturated FAMEs (*n*-C_20:0_ to *n*-C_28:0_) detected in SSJ4 can be indicative of microalgae (Volkman *et al.,* 1989), terrigenous plant origins, or early diagenetic selective preservation (Haddad *et al.,* 1992). The long-chain FAMEs identified in this sample exhibit a strong odd-over-even carbon number preference, which is consistent with a terrigenous origin likely in the form of cuticular waxes (Eglinton and Hamilton, 1967), and are consistent with previous results using the Bligh and Dyer (1959) method, a standard laboratory protocol used for lipid extractions (Johnson *et al.,* manuscript submitted for publication).

#### 4.2.5. Iron Mountain, California, iron sulfide

The PS5P pyrite sample presented unique results and challenges in these experiments. The sample is almost completely composed of pyrite (FeS_2_), with very little quartz present. Only 6 FAMEs below *n*-C_13:0_ were detected when using either of the pyrolysis methods, and the *n*-C_19:0_ internal standard was either not present (in 35°C min^−1^ pyrolysis ramp) or present at a very low concentration (in 500°C flash pyrolysis). FAMEs may not have been well generated and transferred to the column due to the majority of pyrite decomposition beginning over 446°C (Yani and Zhang, 2010), which was not met with the SAM-like pyrolysis used here and was only exceeded with the 500°C flash pyrolysis method. Any organics bound within the pyrite may have just begun to be released during pyrite decomposition and so were not fully methylated prior to transfer to the column. For comparison, the Fe(III) oxide mineral goethite decomposes between 290°C and 330°C (Govaert *et al.,* 1976; Földvári, 2011), liberating putative macromolecules for reaction with TMAH at the temperatures achieved with the SAM-like and flash pyrolysis methods. Future benchtop and flight experiments should therefore consider mineral decomposition temperatures prior to establishing a maximum pyrolysis temperature.

#### 4.2.6. Icelandic siliceous sinter

The inactive near-vent sinter deposit at Gunnuhver demonstrated a decrease in FAME detection at depth with both the SAM-like method (from 6 to 4 FAMEs detected) and the flash pyrolysis method (from 13 to 11 FAMEs detected). With the SAM-like method, the *n*-C_8:0_, *n*-C_14:0_, and *n*-C_18:0_ FAMEs were lacking in the subsurface relative to the surface samples, indicating FAME degradation in the subsurface. With the flash pyrolysis method, the *n*-C_17:0_ and *cis*-C_18:1_ FAMEs were not detected in the subsurface sample, which may indicate some early diagenetic processing of the sinter which will decrease the concentration of preserved MUFAMEs.

The active vent deposit at Hveravellir demonstrated a similar decrease in FAME detection at depth with both the SAM-like method (from 14 to 11 FAMEs detected) and the flash pyrolysis method (from 17 to 10 FAMEs detected). In the SAM-like method, the *n*-C_6:0_, *cis*-C_16:1_, and *cis*-C_18:1_ FAMEs were lacking in the subsurface relative to the surface samples. The flash pyrolysis method yielded the *cis*-C_16:1_, *n*-C_17_, *cis*-C_17:1_, *cis*-C_18:1_, and C_18:2_ FAMEs in the surface sample (*cis* versus *trans* in the C_18:2_ PUFAME could not be resolved based on retention time of comparable PUFAMEs in the Supelco FAME standard). The presence of C_18:2_ can be indicative of cyanobacterial communities (Kelly and Scheibling, 2012) or fungi (Cantrell *et al.,* 2006) and is a reasonable expectation for a surface-exposed hot spring environment. In the flash pyrolysis method, the *n*-C_6:0_, *n*-C_13:0_, *cis*-C_16:1_, *n*-C_17:0_, *cis*-C_17:1_, *cis*-C_18:1_, and C_18:2_ FAMEs were not detected in the subsurface sample, again indicative of some early diagenetic processing of the sinter. The *n*-C_20:0_ and *n*-C_22:0_ FAMEs, indicative of algae or terrestrial plants (O'Reilly *et al.,* 2017), were also detected in both Hveravellir vent samples by using the flash pyrolysis method.

#### 4.2.7. Bahamian carbonates

Both the SAM-like pyrolysis and flash pyrolysis of the modern carbonate ooid sample yielded FAME results that represent a small fraction of the lipids present in these ooids (O'Reilly *et al.,* 2017). Using an off-line solvent extraction technique, O'Reilly *et al.* (2017) identified monocarboxylic fatty acids as the major lipid class, followed by methyl-branched and β-hydroxy fatty acids, dicarboxylic acids, hopanoic acids, monoethers, alkanes, hopanes, and steranes as carbonate-bound lipids in these modern Bahamian ooids.

Sample CIMO contained 5 detectable FAMEs below *n-*C_17:0_, and these results exclusively represented even carbon number FAMEs. The flash pyrolysis method yielded 17 FAMEs ranging from *n-*C_8:0_ to *n-*C_22:0_, including the MUFAMEs *cis*-C_14:1_, *cis*-C_16:1_, and *cis*-C_18:1_. The even-over-odd carbon number preference of FAMEs < C_20:0_ indicates contributions from a modern microbial community. Although the MUFAMEs were a very minor component of the bound FAME pool in the ooid grains, they were far more abundant in the biofilm attached to the surface of the ooid grains (O'Reilly *et al.,* 2017), and the MUFAME signal detected here is likely from the surface biofilm algae and bacteria.

#### 4.2.8. Organic-rich shale

The even-over-odd carbon number preference of FAMEs > C_18:0_ in sample MES is consistent with microbial alteration of algal detritus within the Messel Shale (Elias *et al.,* 1997). Long straight-chain fatty acids are most consistent with terrestrial plant (C_24_ to C_34_) and algal origins (C_20_ to C_24_) (O'Reilly *et al.,* 2017), and the lipids in the Messel Shale have a demonstrated algal origin (Goth *et al.,* 1988).

#### 4.2.9. *n*-C_19:0_ internal standard anomaly

The *n-*C_19:0_ internal standard was injected into each sample immediately prior to analysis to determine the reaction efficiency and was successfully detected in all SAM-like pyrolysis analyses with the notable exception of the iron-bearing samples. Comparably longer-chain FAMEs, such as *n*-C_18:0_, are detected in select iron-bearing samples (SSJ3 and SSJ4), but FAME detection in most iron-bearing samples ended at *n*-C_16:0_ or shorter chains. It is possible in these cases that the long-chain FAMEs, including *n-*C_19:0_, are breaking down in the pyrolysis oven. In comparison, when subjected to the 500°C flash pyrolysis method, the *n-*C_19:0_ FAME was detected in all samples, with the peak in the PS5P pyrite sample being extremely small.

SAM-like pyrolysis of both the pyrite and the acidic Fe(III)-bearing samples did not yield long-chain FAMEs. However, SAM-like pyrolysis of the circumneutral Fe(III)-bearing samples did yield long-chain FAMEs. It appears that the samples formed in acidic environments may be undergoing a reaction with the strongly alkaline TMAH in the pyrolysis oven. This and/or some other synergistic processes could be retaining or destroying the long-chain FAMEs in these samples at slow SAM-like pyrolysis ramps. The results suggest a link between the loss of long-chain FAMEs, the presence of iron minerals formed in acidic environments, and the 35°C min^−1^ SAM-like ramp, but a clear mechanism for the loss of the long-chain FAMEs is not yet elucidated.

#### 4.2.10. Comparison of background (“TMAH blank”) to sample signal

The identification of FAMEs in this work was done in part by performing a “TMAH blank” run before each sample analysis. As with the samples, a cup with 4 μL of TMAH was analyzed through the full pyrolysis and GC-MS programs to determine the background level of FAME contamination and possible carryover from previous samples. Each “TMAH blank” spectrum was subtracted from the subsequent sample spectrum to remove any potential carryover signal. No data is reported in which the background (“TMAH blank”) spectrum was higher than the sample spectrum, with the assumption being that the sample signal was not elevated enough to be confidently identifed above background levels.

To quantify differences between the two spectra for each FAME peak, the percentage that the background spectrum represents relative to the signal spectrum was calculated. Although all data presented in Table 4 represent signal elevated above background, the calculation in Supplementary Table S2 serves as an indicator that the signal is either greatly elevated above background (0% of the background is included in the signal) or is very similar to background (up to 100% of the background is included in the signal). Therefore, lower percentages represent a greater signal-to-noise ratio and indicate more confidence that the FAME peak identified is native to the sample.

In general, the saturated FAME, MUFAME, and PUFAME signal in most samples was greatly elevated over background values (as indicated in Supplementary Table S2 with values closer to 0%). In some cases, the background/sample signal ratio varied greatly (*e.g.,* flash pyrolysis PS5G and CC). In these situations, most FAME peaks were greatly elevated above background, with one or a few FAME peak values (mostly the very low or high molecular weight FAMEs) being more similar to the background levels. Samples OCM and PS5P in particular exhibit sample signal values that were quite similar to background values. As the instrument background was always carefully lowered before each run with a series of high-temperature bake-out sequences, blank analyses, and “TMAH blank” flushes, these results indicate that FAME signals in these two samples are very low and are most likely comparable with instrument background.

#### 4.2.11. Comparison of 500°C flash pyrolysis and 35°C min^−1^ ramp pyrolysis methods

The SAM-like pyrolysis employs a 35°C min^−1^ ramp to 400°C, which took *ca.* 10 min to reach maximum temperature, while the flash pyrolysis method used an instantaneous 500°C flash pyrolysis step held for 1 min. Samples treated with the flash pyrolysis step almost always yielded a greater number and diversity of FAMEs than the SAM-like 35°C min^−1^ ramp method. This was likely due to several factors: the gradual ramp at 35°C min^−1^ generates radicals, and the temperature ramp may limit the optimal time for reaction of TMAH with the solid sample. The flash pyrolysis step is likely more efficient in reacting TMAH with macromolecules in the solid sample and transferring the eluent to the column. The flash pyrolysis step generated water, which may serve as a carrier for the eluent and facilitate more efficient transfer to the column. In addition, in flash pyrolysis the sample molecules are released simultaneously to the TMAH as it is vaporized, as opposed to in ramp pyrolysis in which some of the TMAH may have been lost by the time the sample reaches temperatures at which the minerals will degrade and release organics, or temperatures at which the organics vaporize. These factors in concert may limit the number and diversity of FAMEs produced with a 35°C min^−1^ pyrolysis ramp.

#### 4.2.12. Biotic and abiotic hydrocarbon generation and implications for fatty acid detection

The capability of SAM to detect short, medium, and long-chain FAMEs and determine any carbon number preference is critical for determining the provinance of fatty acids on Mars. Biotically generated fatty acids may have characteristic branching or unsaturations that can be used to identify the organism (or organisms) that generated the fatty acid (see Table 5). An even-over-odd carbon number preference is often correlated as a product of bacteria and other microbes in recent to Miocene-aged sediments (Nishimura and Baker, 1986; Grimalt and Albaiges, 1987) and may indicate the presence of a recent microbial community. Depending on the environment, this even-over-odd preference can reflect reductive processes altering lipid compounds, or microbial alteration of algal detritus on Earth (Elias *et al.,* 1997).

In contrast, some aliphatic and aromatic hydrocarbons may be generated abiotically. One such pathway enlists Fischer-Tropsch-type reactions, in which iron from a meteoric source serves as the catalyst to generate lipids from carbon monoxide and hydrogen (Gelpi *et al.,* 1970). In these senarios, saturated and unsaturated hydrocarbons may be generated that range from *n*-C_2:0_ to >*n*-C_35:0_, but with no carbon number preference (*e.g.,* the carbon number preference index = 1.0 [Bray and Evans, 1961; Gelpi *et al.,* 1970; McCollom *et al.,* 1999]). Interplanetary dust particles or cometary infall may undergo these reactions on Mars to generate a steady signal of aliphatic hydrocarbons that are detectable with the SAM TMAH experiment.

It is important to note that the carbon chain length and carbon number preference are not always diagnostic of biotic versus abiotic genesis. Short-chain carboxylic acids may represent metabolic products not incorporated into membranes, and as discussed above, Fischer-Tropsch-type reactions can generate long-chain carboxylic acids. A carbon number preference may be more diagnostic of abiotic versus biotic processes, but the even-over-odd or odd-over-even preference may differ from trends observed on Earth. The even-over-odd expression in lipid chains, a result of enzymatic biochemistry, reflects monomer addition of a 2-C subunit. In contrast, isoprenoid lipids are built by 5-C isoprene subunits. It is the addition of subunits by the enzymatic activity of organisms that is associated with the lipid carbon number preference biosignature (Summons *et al.,* 2008; Georgiou and Deamer, 2014), and it is possible that extraterrestrial life, if present, may build lipid structures of 2-C, 3-C, 4-C, 5-C, and so on, subunits. Therefore, studies such as the one presented here are vital to further resolving the abiotic versus biotic genesis of fatty acids detected with the *in situ* TMAH experiment.

Lastly, the presence of perchlorates on Mars may dramatically affect the preservation and detection of any organics. The Viking life-detection experiments were intially interpreted to show a dearth of native organics (Biemann *et al.,* 1976, 1977). These results have recently been reinterpreted to reveal that the detected organics chloromethane and dichloromethane are the result of martian organics reacting with perchlorates during heating (Navarro-González *et al.,* 2010; Guzman *et al.,* 2018). Similar concerns exist for analyzing samples with the GC-MS on SAM—chloromethane and chlorobenzene have been detected with SAM with the source attributed to martian perchlorate reactions with the MTBSTFA derivitization reagent (Glavin *et al.,* 2013), and chlorobenzene and C_2_ to C_4_ dichloroalkanes detected are attributed to native martian organics reacting with martian perchlorates (Freissinet *et al.,* 2015). Ongoing work to reduce the effects of perchlorates *in situ* with SAM has proven effective (Freissinet *et al.,* 2018), and continued optimization of SAM's TMAH experiment includes assessing the impact of perchorates on fatty acid detection (Buch *et al.,* 2018). Although chlorine is widespread across Mars (Keller *et al.,* 2006), oxychlorine compounds may not be homogenously distributed, as recent findings from the Curiosity rover indicate that oxychlorine compounds have not been identified since before the Oudam drill site in the Murray formation (elevation -4436 m) (Archer *et al.,* 2018). Although the disappearance of oxychlorine compounds does not correlate with a decrease in elemental Cl, it indicates differences in the types of chlorinated compounds present. A lower perchlorate concentration in a given sample may allow for better organics detection and fewer chlorinated compounds in future SAM analyses. Additionally, if perchlorates are present and affect the detection of fatty acids, information about the molecular weights of the organics generated can still be used to distinguish between likely contamination (only low molecular weight organics) and likely indigenous organics (high molecular weight or a combination of high and low molecular weights) (Sephton *et al.,* 2014).

#### 4.2.13. Strengths and limitations of the SAM instrument and paths forward for future missions

Many parameters can be modified in SAM to optimize GC-MS analyses. These include cup and sample preheat (*e.g.,* no treatment to cup preheat at 200–250°C to sample boil-off up to *ca.* 320°C), pyrolysis temperature ramp (35°C min^−1^ up to *ca.* 70°C min^−1^, with final temperatures *ca.* 960°C), and GC oven temperature ramp (5–15°C min^−1^, with final temperatures *ca.* 250°C). See the work of Freissinet *et al.* (2015) for details on SAM flight model operational parameters. These parameters will be tested on the highest-fidelity SAM-like systems at NASA Goddard Space Flight Center to optimize the TMAH experiment for operation on Mars.

As discussed previously, long-chain FAMEs that were detected with the flash pyrolysis method were often missing when analyzed with the SAM-like pyrolysis method. This lack of long-chain FAMEs may have been due to the slow ramp of the pyrolysis oven, which may have broken down long-chain FAMEs to shorter chains which are then readily detected. This side effect of the slower ramp method may not affect *in situ* SAM performance, as initial benchtop analyses with a spare GC5 column suggest that smaller and shorter-chain molecules are more likely to be detected with SAM at nominal operating conditions (Millan *et al.,* 2016). In the Millan *et al.* (2016) experiments, the largest molecule detected with flight spare GC5 column is naphthalene (molar mass = 128 g mol^−1^), and the largest molecule detected with the flight model GC5 column is biphenyl (molar mass = 154 g mol^−1^).

**FIG. 9.**
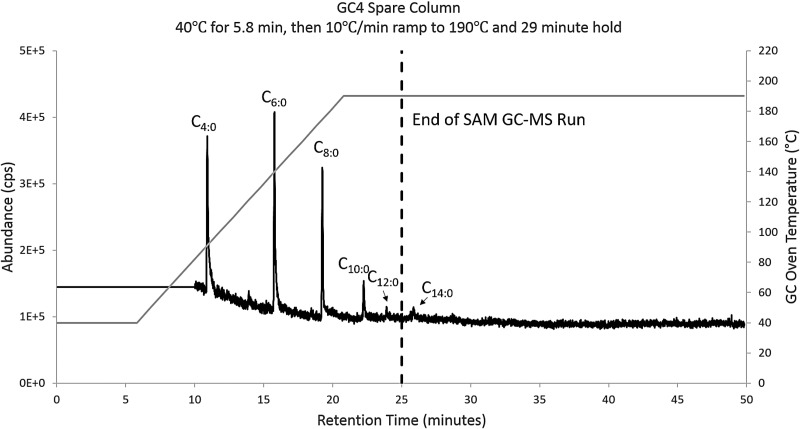
Total ion chromatogram of the Supelco 37 FAME standard analyzed on the SAM spare GC4 column. Analysis using a 5.8 min hold at 40°C and 10°C min^−1^ GC oven ramp. All the species after the 25 min of the SAM GC run cannot be detected under nominal operating conditions.

Of SAM's six GC columns, GC4, GC1, and until recently GC5, are the most commonly used. Split runs have been developed that allow a sample to be put on two columns during one pyrolysis run, effectively getting two analyses for (almost) the energy and resource costs of one analysis. The TMAH experiment on SAM will likely be conducted using a split between GC4, which has retention times similar to GC5 but cannot exceed ∼190°C, and GC1, which does not have a thermal conductivity detector (TCD) and can therefore be heated to ∼250°C. To determine the largest FAME that could be detected with SAM, the retention time of the Supelco 37 FAME standard was determined on the flight spare GC4 column using SAM-like flow and GC oven ramps. The largest FAME detected with a 10°C min^−1^ GC ramp was C_12:0_ (Fig. 9). The SAM GC4 run ends at 25 min due to a flight column temperature limit and a short ∼3 min hold. New and creative approaches may be developed that enable SAM to detect longer-chain FAMEs and determine the organic carbon sources and load on the martian surface and near-surface.

Other considerations for the *in situ* experiment include (1) the detection of other organic molecules beyond FAMEs associated with biologic origins and (2) the potential for radiation-induced decomposition of fatty acids. A complete summary of these topics is beyond the scope of this work and constitutes ongoing research in the field. In general, many other organic molecules may undergo thermochemolysis and be detected during the *in situ* experiment. For example, dicarboxylic acids such as those found in the Krebs cycle are detectable with this experiment and indeed have been previously detected in the Murchison meteorite (Lawless *et al.,* 1974). The potential for radiation-induced decomposition of fatty acids is also important to note for this work. Radiolytic products of fatty acids tend to lose one carbon atom, which would change the biological fingerprint of the even-over-odd carbon number preference (Kim *et al.,* 2004). However, any preference may be used to differentiate the FAME profile from an abiotic pattern with no carbon number preference.

The ExoMars rover will carry the MOMA instrument, which is also capable of detecting organic molecules with derivitization and thermochemolysis py-GC-MS experiments. MOMA will be able to ramp the pyrolysis oven at >200°C min^−1^ (from thermal vacuum), achieving a much faster ramp than SAM and perhaps increasing the likelihood of detecting martian FAMEs, if present. Lessons learned from the operation of the wet chemistry experiments on SAM will be used to further optimize these experiments on MOMA.

## 5. Conclusions

This work represents the first analyses of a suite of Mars-analog samples using the TMAH experiment under select SAM-like conditions. The following experiment parameters were explored: sample exposure time to TMAH, TMAH reactions with MTBSTFA, and loss of the TMAH solvent methanol prior to sample pyrolysis. Samples may be exposed to TMAH and left to react in the SAM oven for up to 92 h and still yield detectable FAMEs when pyrolyzed. The MTBSTFA vapor known to be present in the SAM SMS will likely react somewhat with the *in situ* sample, yielding mixed methylated and silylated products that may increase the possibility of detecting fatty acids with SAM. Lastly, the predicted evaporation of MeOH from the TMAH/MeOH mixture will proceed slowly enough at the low operating temperatures and pressures on Mars that it should not significantly affect the *in situ* experiment.

Results from analog sample analyses demonstrated that fatty acids are readily methylated by TMAH to FAMEs and made detectable via py-GC-MS using a SAM-like pyrolysis ramp. Analog samples included iron oxyhydroxides/-oxyhydroxysulfates, mixed iron oxide/oxyhydroxides and clay minerals, iron sulfides, siliceous sinter, carbonates, and shale. The TMAH experiments generally performed well under SAM-like pyrolyzer ramp conditions when organics were present and/or preserved in high concentrations, present from modern systems, and/or more preserved in circumneutral mixed mineralogy environments, although the experiments were capable of detecting FAMEs in all samples tested, albeit with a lower abundance and variety of FAMEs. Analog samples were also tested with a flash pyrolysis method meant to reveal a more realistic representation of the organics loads in the analog samples. These flash pyrolysis experiments generated detectable FAMEs with a much higher abundance and greater variety of FAMEs, with results that were consistent with the SAM-like experiments.

Several unexpected results from the SAM-like and flash pyrolysis methods are applied to the continued optimization of the SAM thermochemolysis experiment. In the SAM-like 35°C min^−1^ pyrolysis ramp experiments, long-chain FAMEs, including the *n*-C_19:0_ internal standard, were not consistently detected. The slow ramp at 35°C min^−1^ generates radicals and drives off the TMAH, and may have limited the time for reaction with the solid sample. Iron sulfide presented a unique challenge to thermochemolysis, and organics were not readily extracted from the pyrite sample in this work. The mineralogy of candidate samples for the *in situ* TMAH experiment will need to be well constrained before the experiment proceeds on Mars. The most promising mineralogies on which to perform the *in situ* experiment from this data set are the clay and mixed iron oxide/clay mineralogies, in which the oxidizing potential of the iron oxides appears to be diminished by the preservation potential of the clay minerals. Fe/Mg smectite clay minerals are present in the path of the Curiosity rover in the “clay-bearing unit” (Bennett *et al.,* 2018) adjacent to the iron oxide-rich Vera Rubin Ridge, and these results indicate that the clay-bearing unit may provide the optimal opportunity to detect fatty acids, if present, in Gale Crater.

The TMAH thermochemolysis experiment on SAM represents a unique opportunity to detect organic molecules bound in macromolecules on Mars. The py-GC-MS-generated FAME profile will be key to determining the genesis of fatty acids on Mars. The results from this study provide a framework for sample selection and experiment optimization for the SAM thermochemolysis experiments, as well as the development of the wet chemistry experiments on the ExoMars MOMA instrument.

## Supplementary Material

Supplemental data

Supplemental data

Supplemental data

## Abbreviations Used

DMF: dimethylformamide
FAMEs: fatty acid methyl esters
GC: gas chromatograph
GC-MS: gas chromatography mass spectrometry, gas chromatograph mass spectrometer
MOMA: Mars Organic Molecule Analyzer
MTBSTFA: *N*-methyl-*N-tert*-butyldimethylsilyl-trifluoroacetamide
MUFAMEs: monounsaturated fatty acid methyl esters
OCM: Organic Check Material
PUFAMEs: polyunsaturated fatty acid methyl esters
py-GC-MS: pyrolysis gas chromatography mass spectrometry
QMS: quadrupole mass spectrometer
SAM: Sample Analysis at Mars
SMS: Sample Manipulation System
TLS: tunable laser spectrometer
TMA: trimethylamine
TMAH: tetramethylammonium hydroxide
TOC: total organic carbon
