# Supplementary material for: Recovery of Fatty Acids from Mineralogic Mars Analogs by TMAH Thermochemolysis for the Sample Analysis at Mars Wet Chemistry Experiment on the Curiosity Rover
Source: Astrobiology. 2019 Mar 27;19(4):522–46. doi: 10.1089/ast.2018.1819 (PMC6459279; doi:10.1089/ast.2018.1819)
Supplement: Supplemental data [file Supp_Data.pdf]

Supplementary Material

Sample Preparation Details

The glassware and tools used to prepare the samples at NASA Goddard Space Flight Center were solvent washed and ashed at 500 °C in air overnight (ca. 12 hours). All rock samples were ground to a fine powder <1 mm in diameter (the maximum size fraction for MSL) in an ashed (at 550 °C) mortar and pestle, homogenized by grinding for >3 minutes, and parsed into aliquots with a solvent-washed scoop in a fume hood. All of the sample powders were stored inside solvent-washed clean Teflon capped glass vials. Teflon caps were rinsed in triplicate with methanol, dichloromethane, and hexane, and left to dry under a fume hood before capping solvent-washed and ashed (at 500 °C) glass vials.

Analog Site Sampling Details

The cryoconite sample was dried under vacuum in a Buchi Rotavapor R-215 for 24 hours before being ground to a fine powder in an ashed (at 550 °C) mortar and pestle.

The Iron Mountain massive sulfide deposit samples were not initially collected using solvent-washed or ashed tools. Therefore large (>29 cm wide) rock samples of goethite (sample PS5G) and pyrite (sample PS5P) from the Iron Mountain gossan were broken open on ashed ultra-high vacuum (UHV) foil with a rock hammer wrapped in UHV foil to access the uncompromised rock interior. The rock interiors were sampled with a solvent-cleaned (MeOH – MeOH/DCM – DCM) drill bit and collected into ashed (at 500 °C) glass vials.

The modern Iron Mountain pipe precipitate samples were collected with a sterile (flamed) chisel and placed into acid-washed glass jars for transport back to the laboratory. These samples were originally collected for biological analyses.

All sinter samples were collected using solvent-washed and ashed tools. Samples were extracted using solvent cleaned (MeOH – MeOH/DCM – DCM) and ashed (at 500 °C) chisels, spoons, and tweezers. Rock hammers were covered in a new piece of ashed (at 500 °C) foil before use. New solvent cleaned and ashed tools were used to collect samples both at the surface and at depth. Samples were placed in solvent washed and ashed (at 500 °C) glass jars for transport back to the laboratory.

The carbonate ooid and shale samples were collected using solvent-washed and ashed tools, handled with solvent-cleaned steel implements and ashed (500 °C) aluminum foil, and powdered in a SPEX 8510 shatterbox. The steel puck mill was thoroughly cleaned, dried and sonicated in methanol and then dichloromethane prior to use. The modern ooids were freeze-dried using a VirTis Benchtop freeze dryer prior to powdering.

Pyrolysis GC-MS Program Conditions

For the TMAH volume to sample mass ratio experiments, the sample was dropped into a 600°C pyrolyzer oven with a 2 minute hold. The inlet program and Tenax ® TA trap started at 15 °C with a 2 minute hold and a 1:10 split. The inlet was ramped at 68 °C min^{-1} to 150 °C and held for 2 minutes, then ramped at 80 °C min^{-1} to 310 °C and held for 3 minutes, then ramped at 100 °C min^{-1} to 250 °C with no hold to mimic the SAM flight instrument GC ramp and optimize the detection of smaller FAMEs of interest with a slower initial GC ramp. The GC column program started at 50 °C and was held for 11 minutes, then ramped at 15 °C min^{-1} to 175 °C with no hold, then ramped at 10 °C min^{-1} to 300 °C and held for 1 minute. The auxiliary transfer line was held at 135 °C.

For the TMAH duration exposure experiments and the TMAH + MTBSTFA experiments, the sample was dropped into a 600 °C pyrolyzer oven with a 2 minute hold. The inlet program and Tenax ® TA trap started at 15 °C with a 2 minute hold and a 1:10 split. The inlet was ramped at 68 °C min^{-1} to 150 °C and held for 2 minutes, then ramped at 80 °C min^{-1} to 310 °C and held at 3 minutes, then ramped at 100 °C min^{-1} to 250 °C with no hold. The GC column program started at 50 °C and was held for 11 minutes, then ramped at 15 °C min^{-1} to 175 °C with no hold, then ramped at 10 °C min^{-1} to 300 °C and held for 10 minutes. The auxiliary transfer line was held at 270 °C.

For the SAM-like pyrolysis ramp and GC column method, the pyrolyzer program started at 50 °C and was ramped at 35 °C min^{-1} to 400 °C with no hold. The inlet program and Tenax ® TA trap started at 15 °C with a 2 minute hold and a 1:10 split. The inlet was ramped at 68 °C min^{-1} to 150 °C and held for 2 minutes, then ramped at 80 °C min^{-1} to 305 °C and held at 3 minutes, then ramped at 100 °C min^{-1} to 250 °C with no hold. The GC column program started at 35 °C and was held for 10.9 minutes, then ramped at 15 °C min^{-1} to 175 °C with no hold, then ramped at 10 °C min^{-1} to 305 °C and held for 1 minute. The auxiliary transfer line was held at 135 °C.

Samples were also analyzed using a 500 °C flash pyrolysis method with the following program to increase the diversity and abundance of detectable FAMEs: the sample was dropped into a 500°C pyrolyzer oven and held for 1 minute. The inlet program and Tenax ® TA trap started at 15 °C with a 2 minute hold and a 1:10 split. The inlet was ramped at 900 °C min^{-1} to 300 °C and held for 2 minutes. The GC column program started at 35 °C and was held for 2.5 minutes, then ramped at 5 °C min^{-1} to 300 °C and held for 5 minutes. The auxiliary transfer line was held at 270 °C.
