# Supplementary material for: Recovery of Fatty Acids from Mineralogic Mars Analogs by TMAH Thermochemolysis for the Sample Analysis at Mars Wet Chemistry Experiment on the Curiosity Rover
Source: Astrobiology. 2019 Mar 27;19(4):522–46. doi: 10.1089/ast.2018.1819 (PMC6459279; doi:10.1089/ast.2018.1819)
Supplement: Supplemental data [file Supp_Table1.pdf]

SM Table S1. FAME relative abundance, CPS, and % difference between sample and blank signal. CPS reported from m/z = 143, except for C_6 , which is m/z = 87, and C_4, which is m/z = 74.

		SS12 modern iron precipitate			PS5G older iron oxides			PS5P older pyrite			CIMO modern ooids		
		Rel Abd	*CPS*	*% difference*	*Rel Abd*	*CPS*	*% difference*	*Rel Abd*	*CPS*	*% difference*	*Rel Abd*	*CPS*	*% difference*
**SAM-like 35°C/min^{-1} pyrolysis ramp**	C4	--	--	--	--	--	--	--	--	--	--	--	--
	C6	--	--	--	present	1399	4.9%	present	1412	8.8%	present	8233	9.6%
	C8	--	--	--	present	16	0.0%	present	54	10.0%	5.97%	374	3.4%
	C9	--	--	--	present	155	14.8%	present	493	4.1%	--	--	--
	C10	present	5059	26.0%	present	156	22.8%	present	111	56.0%	--	--	--
	C11	--	--	--	--	--	--	--	--	--	--	--	--
	C12	present	45141	0.0%	--	--	--	--	--	--	22.86%	2507	3.8%
	C13	--	--	--	--	--	--	--	--	--	--	--	--
	C14	--	--	--	--	--	--	--	--	--	25.64%	1858	4.3%
	C15	present	2436	0.9%	--	--	--	--	--	--	--	--	--
	C16	present	6480	0.2%	--	--	--	--	--	--	45.53%	1079	0.6%
	C16:1	--	--	--	--	--	--	--	--	--	--	--	--
	C17	--	--	--	--	--	--	--	--	--	--	--	--
	C18	--	--	--	--	--	--	--	--	--	--	--	--
	C18:1	--	--	--	--	--	--	--	--	--	--	--	--
	C18:2	--	--	--	--	--	--	--	--	--	--	--	--
	C19 STD	--	--	--	--	--	--	--	--	--	present	121	--
	C20	--	--	--	--	--	--	--	--	--	--	--	--
	C21	--	--	--	--	--	--	--	--	--	--	--	--
	C22	--	--	--	--	--	--	--	--	--	--	--	--
	C23	--	--	--	--	--	--	--	--	--	--	--	--
	C24	--	--	--	--	--	--	--	--	--	--	--	--
	C25	--	--	--	--	--	--	--	--	--	--	--	--
	C26	--	--	--	--	--	--	--	--	--	--	--	--
	C27	--	--	--	--	--	--	--	--	--	--	--	--

	C28	--	--	--	--	--	--	--	--	--	--	--	--
	C29	--	--	--	--	--	--	--	--	--	--	--	--
	C30	--	--	--	--	--	--	--	--	--	--	--	--

**500°C Flash Pyrolysis**		SS12 modern iron precipitate			PS5G older iron oxides			PS5P older pyrite			CIMO modern ooids		
		Rel Abd	*CPS*	*% difference*	*Rel Abd*	*CPS*	*% difference*	*Rel Abd*	*CPS*	*% difference*	*Rel Abd*	*CPS*	*% difference*
	C4	--	--	--	--	--	--	--	--	--	--	--	--
	C5	--	--	--	--	--	--	--	--	--	--	--	--
	C6:1	--	--	--	--	--	--	--	--	--	--	--	--
	C6	--	--	--	--	--	--	present	4149	31.0%	--	--	--
	C7:1	--	--	--	--	--	--	--	--	--	--	--	--
	C7	--	--	--	--	--	--	--	--	--	--	--	--
	C8:1	--	--	--	--	--	--	--	--	--	--	--	--
	C8	--	--	--	present	7	73.1%	present	149	10.8%	present	264	35.6%
	C9:1	--	--	--	--	--	--	--	--	--	--	--	--
	C9	present	2952	1.9%	present	121	5.5%	present	52	84.5%	present	5944	0.8%
	C10:1	--	--	--	--	--	--	--	--	--	--	--	--
	C10	present	1261	3.4%	present	226	17.8%	present	765	10.8%	present	7203	2.6%
	C11:1	--	--	--	--	--	--	--	--	--	--	--	--
	C11	present	1121	13.8%	--	--	--	present	82	61.0%	--	--	--
	C12:1	--	--	--	--	--	--	--	--	--	--	--	--
	C12	present	25783	0.5%	present	69	18.8%	present	98	57.6%	present	5595	2.0%
	C13:1	--	--	--	--	--	--	--	--	--	--	--	--
	C13	--	--	--	--	--	--	--	--	--	present	3190	6.0%
	C14:1	--	--	--	--	--	--	--	--	--	present	3116	4.6%
	C14	present	1263	12.6%	present	113	8.1%	--	--	--	present	11786	0.9%
	C15:1	--	--	--	--	--	--	--	--	--	--	--	--
	C15	--	--	--	--	--	--	--	--	--	present	4430	1.6%
	C16:1	present	945	1.9%	--	--	--	--	--	--	present	2601	2.9%
	C16	present	46544	2.2%	present	270	0.0%	--	--	--	present	19826	3.5%

	C17:1	--	--	--	--	--	--	--	--	--	--	--	--
	C17	--	--	--	--	--	--	--	--	--	present	2937	5.1%
	C18:3	--	--	--	--	--	--	--	--	--	--	--	--
	C18:2	--	--	--	--	--	--	--	--	--	--	--	--
	C18:1	--	--	--	--	--	--	--	--	--	--	--	--
	C18:1	present	542	1.1%	--	--	--	--	--	--	present	1706	6.8%
	C18	present	4377	7.9%	present	59	9.2%	--	--	--	present	18902	3.4%
	C19:1	--	--	--	--	--	--	--	--	--	--	--	--
	C19 STD	present	3593	--	present	345	--	--	--	--	present	318635	--
	C20:1	--	--	--	--	--	--	--	--	--	--	--	--
	C20	--	--	--	--	--	--	--	--	--	present	1923	11.1%
	C21:1	--	--	--	--	--	--	--	--	--	--	--	--
	C21	--	--	--	--	--	--	--	--	--	present	526	14.9%
	C22:1	--	--	--	--	--	--	--	--	--	--	--	--
	C22	--	--	--	--	--	--	--	--	--	present	612	11.2%
	C23	--	--	--	--	--	--	--	--	--	--	--	--
	C24:1	--	--	--	--	--	--	--	--	--	--	--	--
	C24	--	--	--	--	--	--	--	--	--	--	--	--
	C25	--	--	--	--	--	--	--	--	--	--	--	--
	C26	--	--	--	--	--	--	--	--	--	--	--	--
	C27	--	--	--	--	--	--	--	--	--	--	--	--
	C28	--	--	--	--	--	--	--	--	--	--	--	--
	C30	--	--	--	--	--	--	--	--	--	--	--	--

SM Table S1. Continued

SAM-like 35°C/min^{-1} pyrolysis ramp		MES			SSJ2			SSJ3			SSJ4			SSJ5		
		Rel Abd	*CPS*	*% difference*	*Rel Abd*	*CPS*	*% difference*	*Rel Abd*	*CPS*	*% difference*	*Rel Abd*	*CPS*	*% difference*	*Rel Abd*	*CPS*	*% difference*
	C4	--	--	--	present	2376	19.0%	--	--	--	--	--	--	--	--	--
	C6	present	nd	0.0%	present	2642	16.5%	--	--	--	present	2434	20.6%	--	--	--
	C8	0.00%	3171	0.3%	present	155	9.9%	--	--	--	present	169	27.2%	--	--	--
	C9	0.40%	28854	0.1%	present	452	21.0%	--	--	--	present	1042	34.3%	--	--	--
	C10	5.10%	79186	0.0%	present	548	0.0%	--	--	--	present	839	19.6%	--	--	--
	C11	3.70%	44811	0.0%	--	--	--	--	--	--	present	736	10.2%	--	--	--
	C12	0.60%	45446	0.0%	present	122	11.6%	--	--	--	present	1297	0.0%	--	--	--
	C13	1.00%	51675	0.0%	--	--	--	--	--	--	present	421	3.0%	--	--	--
	C14	0.80%	70088	0.0%	--	--	--	present	58	20.6%	present	1102	1.0%	--	--	--
	C15	0.90%	48284	0.1%	--	--	--	--	--	--	present	513	1.5%	--	--	--
	C16	5.20%	156928	0.0%	--	--	--	--	--	--	present	7534	0.0%	--	--	--
	C16:1	--	--	--	--	--	--	--	--	--	--	--	--	--	--	--
	C17	1.30%	54629	0.0%	--	--	--	--	--	--	--	--	--	--	--	--
	C18	3%	126032	0.0%	--	--	--	present	22	0.0%	present	276	4.2%	--	--	--
	C18:1	--	--	--	--	--	--	--	--	--	--	--	--	--	--	--
	C18:2	--	--	--	--	--	--	--	--	--	--	--	--	--	--	--
	C19 std	present	1841662	--	--	--	--	--	--	--	--	--	--	--	--	--
	C20	1.60%	114508	0.0%	--	--	--	--	--	--	--	--	--	--	--	--
	C21	1.90%	43558	0.0%	--	--	--	--	--	--	--	--	--	--	--	--
	C22	8.90%	327410	0.0%	--	--	--	--	--	--	--	--	--	--	--	--
	C23	1.70%	73229	0.0%	--	--	--	--	--	--	--	--	--	--	--	--
	C24	31.40%	972210	0.0%	--	--	--	--	--	--	--	--	--	--	--	--
	C25	3.30%	105164	0.0%	--	--	--	--	--	--	--	--	--	--	--	--
	C26	23.10%	381376	0.0%	--	--	--	--	--	--	--	--	--	--	--	--
	C27	6.00%	74164	0.0%	--	--	--	--	--	--	--	--	--	--	--	--

	C28	0.10%	106134	0.0%	--	--	--	--	--	--	--	--	--	--	--	--
	C29	0.10%	27163	0.1%	--	--	--	--	--	--	--	--	--	--	--	--
	C30	0.00%	31352	0.0%	--	--	--	--	--	--	--	--	--	--	--	--

		MES			SSJ2			SSJ3			SSJ4			SSJ5		
		Rel Abd	*CPS*	*% difference*	*Rel Abd*	*CPS*	*% difference*	*Rel Abd*	*CPS*	*% difference*	*Rel Abd*	*CPS*	*% difference*	*Rel Abd*	*CPS*	*% difference*
**500°C Flash Pyrolysis**	C4	present	136128	3.1%	--	--	--	--	--	--	present	6524	26.3%	--	--	--
	C5	present	182016	1.3%	--	--	--	--	--	--	--	--	--	--	--	--
	C6:1	present	10271	12.4%	--	--	--	--	--	--	--	--	--	--	--	--
	C6	present	92656	2.3%	--	--	--	--	--	--	present	4939	9.1%	present	5617	5.6%
	C7:1	present	35512	0.8%	--	--	--	--	--	--	--	--	--	--	--	--
	C7	present	140544	0.7%	--	--	--	--	--	--	--	--	--	--	--	--
	C8:1	present	345	32.5%	--	--	--	--	--	--	--	--	--	--	--	--
	C8	present	2147	12.9%	present	86	15.7%	present	93	16.2%	present	285	5.9%	present	203	32.3%
	C9:1	present	6847	1.7%	--	--	--	--	--	--	--	--	--	--	--	--
	C9	present	37147	0.9%	present	398	4.1%	present	572	0.9%	present	1714	1.7%	present	640	7.4%
	C10:1	present	187816	0.1%	--	--	--	--	--	--	--	--	--	--	--	--
	C10	present	91512	0.4%	present	612	17.4%	present	601	16.2%	present	4384	1.3%	present	528	11.7%
	C11:1	present	39242	0.3%	--	--	--	--	--	--	--	--	--	--	--	--
	C11	present	87592	0.2%	--	--	--	present	349	4.9%	present	2498	3.3%	present	296	10.3%
	C12:1	present	45534	0.5%	--	--	--	--	--	--	--	--	--	--	--	--
	C12	present	103924	0.2%	present	363	4.0%	present	379	7.3%	present	2613	2.2%	present	364	2.7%
	C13:1	present	38126	0.3%	--	--	--	--	--	--	--	--	--	--	--	--
	C13	present	77163	0.2%	--	--	--	present	242	10.4%	present	1861	0.7%	present	294	2.0%
	C14:1	present	51778	0.2%	--	--	--	--	--	--	--	--	--	--	--	--
	C14	present	78635	0.2%	present	344	6.0%	present	745	0.5%	present	2312	0.5%	present	510	11.0%
	C15:1	present	40038	0.2%	--	--	--	--	--	--	--	--	--	--	--	--
	C15	present	84579	0.1%	--	--	--	present	398	5.7%	present	912	3.0%	present	396	4.3%
	C16:1	present	38992	1.4%	--	--	--	--	--	--	--	--	--	--	--	--

	C16	present	123466	0.3%	present	1277	7.6%	present	8631	0.3%	present	7834	0.3%	present	5166	2.1%
	C17:1	present	35012	0.3%	--	--	--	--	--	--	--	--	--	--	--	--
	C17	present	81158	0.2%	--	--	--	present	568	1.9%	present	864	1.0%	present	478	3.8%
	C18:3	--	--	--	--	--	--	--	--	--	--	--	--	--	--	--
	C18:2	--	--	--	--	--	--	--	--	--	--	--	--	--	--	--
	C18:1	--	--	--	--	--	--	--	--	--	--	--	--	--	--	--
	C18:1	present	25980	22.0%	--	--	--	--	--	--	--	--	--	--	--	--
	C18	present	78709	0.9%	present	368	0.0%	present	4365	0.3%	present	4389	0.4%	present	2761	0.5%
	C19:1	present	27703	1.3%	--	--	--	--	--	--	--	--	--	--	--	--
	C19	present	255767	--	present	205	--	present	180095	--	present	1912	--	present	63508	--
	C20:1	present	24213	0.6%	--	--	--	--	--	--	--	--	--	--	--	--
	C20	present	45796	0.8%	--	--	--	--	--	--	present	1624	1.0%	--	--	--
	C21:1	present	21232	1.1%	--	--	--	--	--	--	--	--	--	--	--	--
	C21	present	33050	0.4%	--	--	--	--	--	--	present	767	1.3%	--	--	--
	C22:1	--	--	--	--	--	--	--	--	--	--	--	--	--	--	--
	C22	present	47359	0.3%	--	--	--	--	--	--	present	559	0.0%	--	--	--
	C23	present	21704	0.5%	--	--	--	--	--	--	present	353	0.0%	--	--	--
	C24:1	--	--	--	--	--	--	--	--	--	--	--	--	--	--	--
	C24	present	52901	0.2%	--	--	--	--	--	--	present	132	0.8%	--	--	--
	C25	present	13512	0.6%	--	--	--	--	--	--	present	114	12.3%	--	--	--
	C26	present	24516	0.5%	--	--	--	--	--	--	present	88	0.0%	--	--	--
	C27	present	7643	3.8%	--	--	--	--	--	--	present	83	19.4%	--	--	--
	C28	present	21441	2.0%	--	--	--	--	--	--	--	--	--	--	--	--
	C30	present	3346	nd	--	--	--	--	--	--	--	--	--	--	--	--

SM Table S1. Continued

SAM-like 35°C/min^{-1} pyrolysis ramp

	160726.06.S Gunnuhver vent, recent			160726.06.I Gunnuhver vent, recent			160730.09.S Hveravellir vent, active			160730.09.I Hveravellir vent, active		
	Rel Abd	*CPS*	*% difference*	*Rel Abd*	*CPS*	*% difference*	*Rel Abd*	*CPS*	*% difference*	*Rel Abd*	*CPS*	*% difference*
C4	--	--	--	--	--	--	--	--	--	--	--	--
C6	--	--	--	present	1392	24.1%	present	7539	17.1%	--	--	--
C8	19.50%	136	8.7%		--	--	0.40%	633	9.8%	2.99%	5807	1.2%
C9	28.70%	783	27.2%	50.39%	51	57.5%	5.70%	10043	1.1%	18.25%	14424	3.0%
C10	25.40%	322	30.8%	29.39%	228	29.9%	1.77%	3805	8.0%	1.26%	2776	5.1%
C11		--	--		--	--	0.10%	1479	12.6%	0.97%	1387	13.6%
C12	5.00%	178	14.8%	20.22%	176	0.6%	0.82%	1615	3.0%	0.97%	1480	6.9%
C13		--	--	--	--	--	2.07%	1726	2.5%	0.23%	628	12.8%
C14	20.50%	143	13.3%	--	--	--	7.30%	11791	0.6%	0.94%	3329	2.5%
C15	--	--	--	--	--	--	1.74%	2121	1.0%	0.16%	890	10.9%
C16	--	--	--	--	--	--	0.02%	68504	0.0%	38.67%	40623	0.3%
C16:1	--	--	--	--	--	--	50.50%	437	6.2%	--	--	--
C17	--	--	--	--	--	--	0.43%	2105	1.4%	2.35%	1510	6.1%
C18	0.90%	1	94.1%	--	--	--	0.38%	39784	0.0%	33.21%	59584	0.0%
C18:1	--	--	--	--	--	--	28.76%	953	0.8%	--	--	--
C18:2	--	--	--	--	--	--	--	--	--	--	--	--
C19 std	present	17	--	present	4	--	present	61162	--	present	1068001	--
C20	--	--	--	--	--	--	--	--	--	--	--	--
C21	--	--	--	--	--	--	--	--	--	--	--	--
C22	--	--	--	--	--	--	--	--	--	--	--	--
C23	--	--	--	--	--	--	--	--	--	--	--	--
C24	--	--	--	--	--	--	--	--	--	--	--	--
C25	--	--	--	--	--	--	--	--	--	--	--	--
C26	--	--	--	--	--	--	--	--	--	--	--	--
C27	--	--	--	--	--	--	--	--	--	--	--	--
C28	--	--	--	--	--	--	--	--	--	--	--	--

	C29	--	--	--	--	--	--	--	--	--	--	--	--
	C30	--	--	--	--	--	--	--	--	--	--	--	--

		160726.06.S Gunnuhver vent, recent			160726.06.I Gunnuhver vent, recent			160730.09.S Hveravellir vent, active			160730.09.I Hveravellir vent, active		
		Rel Abd	*CPS*	*% difference*	*Rel Abd*	*CPS*	*% difference*	*Rel Abd*	*CPS*	*% difference*	*Rel Abd*	*CPS*	*% difference*
**500°C Flash Pyrolysis**	C4	--	--	--	--	--	--	--	--	--	--	--	--
	C5	--	--	--	--	--	--	--	--	--	--	--	--
	C6:1	--	--	--	--	--	--	--	--	--	--	--	--
	C6	present	4262	17.7%	present	3025	15.5%	present	6248	12.7%	--	--	--
	C7:1	--	--	--	--	--	--	--	--	--	--	--	--
	C7	--	--	--	--	--	--	--	--	--	--	--	--
	C8:1	--	--	--	--	--	--	--	--	--	--	--	--
	C8	present	231	16.0%	present	84	22.9%	present	646	8.5%	present	1282	5.5%
	C9:1	--	--	--	--	--	--	--	--	--	--	--	--
	C9	present	888	1.1%	present	621	9.3%	present	2684	2.7%	present	1175	5.6%
	C10:1	--	--	--	--	--	--	--	--	--	--	--	--
	C10	present	679	12.5%	present	691	19.4%	present	1591	8.1%	present	1154	4.9%
	C11:1	--	--	--	--	--	--	--	--	--	--	--	--
	C11	present	253	16.8%	present	157	46.8%	--	--	--	--	--	--
	C12:1	--	--	--	--	--	--	--	--	--	--	--	--
	C12	present	276	14.0%	present	419	17.2%	present	1262	7.2%	present	1387	6.0%
	C13:1	--	--	--	--	--	--	--	--	--	--	--	--
	C13	present	294	13.0%	present	199	19.4%	present	648	2.3%	--	--	--
	C14:1	--	--	--	--	--	--	--	--	--	--	--	--
	C14	present	478	3.6%	present	478	10.7%	present	4547	1.8%	present	1638	3.4%
	C15:1	--	--	--	--	--	--	--	--	--	--	--	--
	C15	present	214	8.5%	present	264	18.3%	present	1226	2.5%	present	794	8.4%
	C16:1	--	--	--	--	--	--	present	494	6.4%	--	--	--
	C16	present	5410	15.1%	present	3205	19.6%	present	36854	0.4%	present	18994	10.0%
	C17:1	--	--	--	--	--	--	present	384	17.4%	--	--	--

	C17	present	157	23.4%	--	--	--	present	795	0.0%	--	--	--
	C18:3	--	--	--	--	--	--	--	--	--	--	--	--
	C18:2	--	--	--	--	--	--	present	647	5.4%	--	--	--
	C18:1	present	162	7.4%	--	--	--	present	539	1.8%	--	--	--
	C18:1	--	--	--	--	--	--	--	--	--	--	--	--
	C18	present	1770	0.8%	present	378	25.4%	present	7789	1.3%	present	14438	5.3%
	C19:1	--	--	--	--	--	--	--	--	--	--	--	--
	C19	present	29045	--	present	15331	--	present	5511	--	present	8504	--
	C20:1	--	--	--	--	--	--	--	--	--	--	--	--
	C20	--	--	--	--	--	--	present	248	1.6%	present	368	2.4%
	C21:1	--	--	--	--	--	--	--	--	--	--	--	--
	C21	--	--	--	--	--	--	--	--	--	--	--	--
	C22:1	--	--	--	--	--	--	--	--	--	--	--	--
	C22	--	--	--	--	--	--	present	85	9.6%	present	48	12.7%
	C23	--	--	--	--	--	--	--	--	--	--	--	--
	C24:1	--	--	--	--	--	--	--	--	--	--	--	--
	C24	--	--	--	--	--	--	--	--	--	--	--	--
	C25	--	--	--	--	--	--	--	--	--	--	--	--
	C26	--	--	--	--	--	--	--	--	--	--	--	--
	C27	--	--	--	--	--	--	--	--	--	--	--	--
	C28	--	--	--	--	--	--	--	--	--	--	--	--
	C30	--	--	--	--	--	--	--	--	--	--	--	--
