# Supplementary material for: Recovery of Fatty Acids from Mineralogic Mars Analogs by TMAH Thermochemolysis for the Sample Analysis at Mars Wet Chemistry Experiment on the Curiosity Rover
Source: Astrobiology. 2019 Mar 27;19(4):522–46. doi: 10.1089/ast.2018.1819 (PMC6459279; doi:10.1089/ast.2018.1819)
Supplement: Supplemental data [file Supp_Table2.pdf]

SM Table S2. The percentage that background spectrum represents relative to the signal spectrum for all FAME peaks in each sample. Signal is greatly elevated above background near 0%, or is very similar to background near 100%.

Sample	*SAM-like Pyro Ramp Analysis*	*500°C Flash Pyrolysis Analysis*
CC	0.0 to 1.7%	1.4 to 91.0%
OCM	79.1 to 96.1%	71.4 to 89.9%
PS5G	0.0 to 22.8%	0.0 to 73.1%
SS12A	0.0 to 2.6%	0.5 to 13.8%
SSJ5	na	0.5 to 32.3%
SSJ2	0.0 to 21.0%	0.0 to 17.4%
SSJ3	0.0 to 20.6%	0.3 to 16.2%
SSJ4	0.0 to 34.3%	0.0 to 26.3%
PS5P	4.1 to 56.0%	10.8 to 84.5%
IC160726.06.S	8.7 to 94.1%	0.8 to 23.4%
IC160726.06.I	0.6 to 57.5%	9.3 to 46.8%
IC160730.09.S	0.0 to 17.1%	0.0 to 17.4%
IC160730.09.I	0.0 to 13.6%	2.4 to 12.7%
CIMO	0.6 to 9.6%	0.8 to 35.6%
MES	0.0 to 0.3%	0.1 to 32.5%
